# Chemical Composition and *in Vitro* Evaluation of the Antioxidant and Antimicrobial Activities of *Eucalyptus gillii *Essential Oil and Extracts

**DOI:** 10.3390/molecules17089540

**Published:** 2012-08-09

**Authors:** Dorsaf Ben Hassine, Manef Abderrabba, Yan Yvon, Ahmed Lebrihi, Florence Mathieu, François Couderc, Jalloul Bouajila

**Affiliations:** 1 Laboratoire des IMRCP UMR CNRS-5623, Université Paul-Sabatier, 118 route de Narbonne, Toulouse F-31062, France; 2 Laboratoire de Physicochimie des Matériaux, IPEST, La Marsa 2070, Tunisia; 3 LGC UMR 5503 (CNRS/INPT/UPS), 1 Avenue de l'Agrobiopole, Castanet-Tolosan 31326, France

**Keywords:** *Eucalyptus gillii*, essential oil, extracts, GC-MS, antioxidants activity, DPPH, ABTS, antimicrobial activity

## Abstract

In this study, essential oil and various extracts (hexane, petroleum ether, acetone, ethanol, methanol and water) of *Eucalyptus gilii* were screened for their chemical composition, antimicrobial and antioxidant activities. The essential oil chemical composition was analyzed by gas chromatography-mass spectrometry (GC-MS) and gas chromatography-flame ionization detection (GC-FID), respectively. Thirty four compounds were identified, corresponding to 99.5% of the total essential oil. Tannins [104.9–251.3 g catechin equivalent (CE)/Kg dry mass], flavonoids [3.3–34.3 g quercetin equivalent (QE)/Kg dry mass], phenolics [4.7–216.6 g gallic acid equivalent (GAE)/Kg dry mass] and anthocyannins [1.2–45.3 mg cyanidin-3-glucoside equivalent (C3GE)/Kg dry mass] of various extracts were investigated. Free radical scavenging capacity of all samples was determinedt. In the 1,1-diphenyl-2-picrylhydrazyl (DPPH) assay, the IC_50_ of essential oil was 163.5 ± 10.7 mg/L and in the 2,2'-azinobis-3-ethylbenzothiazoline-6-sulphonate (ABTS) assay, it was 94.7 ± 7.1 mg/L. Among the various extracts, the water extract showed the best result (IC_50_ = 11.4 ± 0.6 mg/L) in the DPPH assay which was comparable to vitamin C (IC_50_ = 4.4 ± 0.2 mg/L). The antimicrobial activities were evaluated against different bacterial and fungal strains. Gram positive bacteria were found to be more sensitive to the essential oil and extracts than Gram negative ones. Anthocyanins seem to have a major effect on the growth of *Bacillus subtilis* (R^2^ = 0.79). A significant antifungal activity was observed against the yeast and fungi. Correlations between chemical composition and antioxidant activities were studied and R^2^ values were about 0.96 for the effect of phenolics on the DPPH assay.

## 1. Introduction

In all regions of the World, history shows that medicinal plants have always held an important place. These plants contain essential oils and other substances that can be used in foods (aromas), perfumery (aromatic molecules), aromatherapy, herbal medicines (active) or cosmetics (substances treating skin and hair). Only few hundred, among the countless aromatic species identified in Nature, are used on a commercial scale, so a significant number of these plant resources remain untapped. It has been shown that essential oils obtained from different species of plants are responsible for the antimicrobial effects of spices and herbs that are used to increase the shelf life of foods [1].

Essential oils, currently used as food flavorings, could therefore serve as food preservatives, especially since they are mostly classified Generally Recognized As Safe (GRAS) or approved as food additives by the U.S. Food and Drug Administration [2]. Essential oils have broad spectrum of activity against different bacterial and fungal strains [3]. Furthermore, their antimicrobial activity is mainly based on their chemical composition, in particular the nature of their main volatile components [3,4,5,6]. These medicinal plants are distributed worldwide, for example in Tunisia, where the climatic conditions are favorable for wild, cultivated and introduced plants. The value of these natural resources can have significant economic benefits for the country.

Eucalyptus is a native Australian tree. It is represented by more than 900 species [7]. It has been introduced worldwide, including in Tunisia. Eucalyptus is mainly cultivated for its timber, pulp and essential oils that present medicinal properties and therapeutic uses. It is considered an important source of essential oils used in traditional medicine. Eucalyptus essential oil is used to relieve head colds, rheumatism, muscular pain, and as an expectorant in cases of bronchitis [8].

Our interest has focused on one (not studied) species of the genus Eucalyptus which can serve as raw material for extraction of essential oils and various extracts. Our choice is based on the virtues of this plant. To our knowledge no study has been undertaken on leaf extracts of *E. gillii*. In the present work, we analyzed qualitatively and quantitatively the constituents of *E. gillii* leaves essential oil and various extracts. Antimicrobial and antioxidant activities of the volatile fraction and various extracts from *E. gillii *were also investigated. Correlations between chemical composition and biological activities were studied.

## 2. Results and Discussion

### 2.1. Chemical Composition

#### 2.1.1. Essential Oil

The yield of *E. gillii *leaves essential oil was 2.3% (w/w relative to dry material weight) with a pale yellow color and a persistent odor. Comparing our result to those obtained by Jaimand *et al*. [9], the yield of this species cultivated in Iran was about 2.4%. Although the differences in geographic, climatic and ecologic parameters, no significant variability in essential oil content was observed.

The components of *E. gillii* essential oil have been determined by GC-FID and GC-MS analysis. Thirty-four compounds were identified (Table 1), corresponding to 99.5% of the total essential oil. The major components were 1,8-cineole (43.8%), *p*-cymene (14.2%) and α-pinene (10%). 1,8-Cineole was abundant in all species and these results are in good agreement with those reported by Giamakis *et al*. [10]; (Eucalyptus essential oil is in the range 20%–90%).

**Table 1:** Chemical composition of essential oil from *E. gillii* leaves.

N°	RI	Compounds	%Area
1	936	α-pinene	10.0
2	951	α-fenchene	0.2
3	1025	*p*-cymene	14.2
4	1028	limonene	1.4
5	1030	1,8-cineole	43.8
6	1033	β-phellandrene	0.1
7	1057	γ-terpinene	0.2
8	1086	α-terpinolene	0.2
9	1105	fenchol	0.1
10	1138	*trans*-2-caren-4-ol *	0.1
11	1140	*cis*-sabinol	5.2
12	1145	*trans*-verbenol	0.1
13	1166	borneol	0.3
14	1168	pinocarvone	2.6
15	1195	myrtenal	0.2
16	1202	myrtenol	0.2
17	1208	verbenone	0.5
18	1237	pulegone	0.1
19	1237	cuminaldehyde	0.2
20	1280	piperitone	0.3
21	1288	*p*-cymen-7-ol	0.1
22	1388	β-bourbonene	0.5
23	1516	α-selinene	3.1
24	1527	calamenene	0.1
25	1560	germacrene B	1.1
26	1576	spathulenol	4.1
27	1578	globulol	0.4
28	1590	viridiflorol	0.5
29	1596	guaiol	1.0
30	1648	γ-eudesmol	3.6
31	1650	α-cadinol	1.2
32	1651	β-eudesmol	3.2
33	1805	nootkatone *	0.2
34	nd	dihydroumbellulone	0.1
		Total	99.5
		Monoterpene hydrocarbons	12.2
		Oxygenated monoterpenes	53.6
		Sesquiterpenes hydrocarbons	4.9
		Oxygenated sesquiterpenes	14.2
		Others	14.7

*: Tentative identification supported by good match of MS spectra; nd: Not determined; RI: retention index.

The essential oil consists of oxygenated monoterpenes (53.6%), oxygenated sesquiterpenes (14.2%), monoterpene hydrocarbons (12.2%), sesquiterpene hydrocarbons (4.9%) and other components (14.7%). No data was reported in the literature regarding the chemical composition of *E. gillii* essential oil, but we compared it to other species growing in the same region. For example, Naceur *et al*. [11] indicates that the major components of *E. oleosa* leaves essential oil were α-pinene 12.3%, limonene 12.1% and 1,8-cineole 26.1%. Comparatively, *E. gillii *essential oil from central Tunisia presents a higher amount of 1,8-cineole 43.8% and a lower amount of limonene 1.4%, whereas the amount of α-pinene seems to be the same 10%. This result represents the major finding of our study. The qualitative and quantitative analysis showed variability in the essential oil extracted from *E. gillii* leaves. The chemical composition of essential oil is affected by several factors such as species, geographical location, harvest time, plant part used and isolation method. This variety is different from other *Eucalyptus *in terms of chemical composition of essential oils, which makes the work interesting.

#### 2.1.2. Various Extracts

Extraction yields of various *E. gillii* extracts are presented in Table 2. Hexane extract has the highest yield (30.7%), followed by ethanolic extract (9.0%), methanolic extract (6.4%), then water extract (5.3%) and acetone extract (5.2%). Petroleum ether extract presented only 0.3% yield.

No data relative to our plant has been found in the literature. Amakurra *et al*. [12] used commercial *Eucalyptus* leaves from the Japan Food Additive Association, and the yield of *n*-hexane extract was 6.3%, however the hexane extract of *E.* *gillii* leaves is about 30.7%. According to the authors, the *Eucalyptus* product (50 g) was successively extracted with *n*-hexane (50 mL, four times), ethyl acetate (50 mL, four times) and *n*-butanol (50 mL, four times). Manwhile, Li *et al*. [13] indicate that the hexane yields of Tasmanian *E.* *globulus* leaves ranged from 0.2 to 1.5% dry weight. The yield of *E.* *gillii* acetone leaves extract (5.2%) is more important than the *E.* *globulus* acetone leaves extract (0.2%) obtained by Rahman *et al*. [14]. We noticed that the yield of *E.* *globulus* hot water leaves extract is about 13.58% for Japanese species, obtained by Hasegawa *et al*. [15]; however, for our *E.* *gillii* cold water leaves extract, the yield is about 5.3%.

**Table 2:** Extraction yields (%) of essential oil and various extracts of *E. gillii.*

Samples	Yield (%)
Essential oil	2.3 ± 0.1 ^a^
Hexane	30.7 ± 0.3 ^b^
Petroleum ether	0.3 ± 0.0 ^c^
Acetone	5.2 ± 0.0 ^d^
Ethanol	9.0 ± 0.2 ^e^
Methanol	6.4 ± 0.0 ^d^
Water	5.3 ± 0.0 ^d^

Values within rows with different superscripts (a–e) were significantly different (*p* < 0.05); ±: Standard deviation.

The chemical composition of the various *E. gillii* extracts is summarized in Table 3. For phenolics, water extract was the most rich (216.6 GAE g/Kg dry mass), followed by ethanol extract (143.4 GAE g/Kg dry mass), methanol extract (143.2 GAE g/Kg dry mass), acetone (53.7 GAE g/Kg dry mass) and finally petroleum ether extract (4.5 GAE g/Kg dry mass). No phenolics were found in the hexane extract.

**Table 3:** Chemical composition of *E. gillii *extracts.

Extracts	Phenolics (GAE) ^a^	Tannins (CE) ^a^	Flavonoids (QE) ^a^	Anthocyanins (C3GE) ^b^
Hexane	nd ^a^	152.2 ± 1.4 ^a^	4.0 ± 0.1 ^a^	45.3 ± 0.1 ^a^
Petroleum ether	4.5 ± 0.0 ^b^	133.5 ± 0.8 ^b^	3.3 ± 0.1 ^b^	43.4 ± 0.2 ^a^
Acetone	53.7 ± 0.1 ^c^	104.9 ± 1.4 ^c^	27.6 ± 0.4 ^c^	4.7 ± 0.2 ^b^
Ethanol	143.4 ± 0.1 ^d^	148.6 ± 1.6 ^a^	34.3 ± 0.1 ^d^	nd ^c^
Methanol	143.2 ± 0.7 ^d^	251.3 ± 0.7 ^d^	23.9 ± 0.3 ^e^	1.3 ± 0.0 ^d^
Water	216.6 ± 0.4 ^e^	231.7 ± 0.8 ^e^	14.4 ± 0.2 ^f^	1.2 ± 0.0 ^d^

^a^: g/Kg dry mass; ^b^: mg/Kg dry mass; nd: not detected; Values within columns with different superscripts (a–f) were significantly different (*p* < 0.05); ±: Standard deviation.

The amount of total tannins varied in the different extracts from 104.9 ± 0.7 to 251.3 ± 1.4 Catechin Equivalent g/Kg dry mass. The highest amount of tannins was found in the methanolic extract (251.3 ± 0.7 CE g/Kg dry mass), followed by the water extract (231.7 ± 0.8 CE g/Kg dry mass), then hexane extract (153.2 ± 1.4 CE g/Kg dry mass), ethanol extract (148.6 ± 1.6 CE g/Kg dry mass) and finally acetonic extract (104.9 ± 1.4 CE g/Kg dry mass). 

Flavonoids (3.3–34.3 QE g/Kg dry mass) were also detected in the extracts, the ethanol one (34.3 QE g/Kg dry mass) being the most rich, followed by acetone (27.6 QE g/Kg dry mass) and methanol (23.9 QE g/Kg dry mass). Flavonoids were presented in small amounts compared to the two families mentioned above (phenolics and tannins).

Anthocyanins were also found, but in small quantity compared to the other families, with an average concentration of mg/Kg. Hexane extract (45.3 C3GE mg/Kg dry mass) and petroleum ether extract (43.4 C3GE mg/Kg dry mass) were the richest in anthocyanins.

Variation in the yields of various extracts was attributed to the apolarity of different compounds in the leaves. Such differences have been reported in literature concerning berries of *J. phoenicea * [16]. We didn’t find any data concerning *E. gillii* extracts.

A comparison with the literature showed that the total phenolics was 11.9 mg/g gallic acid equivalents from the commercial *Eucalyptus* leaf extract from the Japan Food Additive Association [12]. It was also cited by Chapuis-Lardy *et al*. [17], that the amount of total phenolics extracted with methanolic solutions from three *Eucalyptus* spp. ranged from 116 to 138 mg Tannin Acid Equivalents (TAE)/g dry matter. Distilled water extracts contained 101 to 126 mg TAE/g dry matter. Our *Eucalyptus* extracts contained more phenolics than those reported in the literature.

The chemical composition of various *Eucalyptus* leaves extracts showed that this species is very rich in phenolics, which are responsible for the antioxidant activity and other beneficial properties of *Eucalyptus* leaves extracts. Phenolic compounds were identified from various *Eucalyptus* leaves, but no such data refers to *E. gillii* leaves. These compounds vary from some species to others, however, the majority of the ones found were not evaluated for their antioxidant activity. That’s why the investigation of these compounds will be of interest to identify any specific molecules which may be responsible for the observed biological activities.

### 2.2. Antioxidant Activity

**Figure 1:**
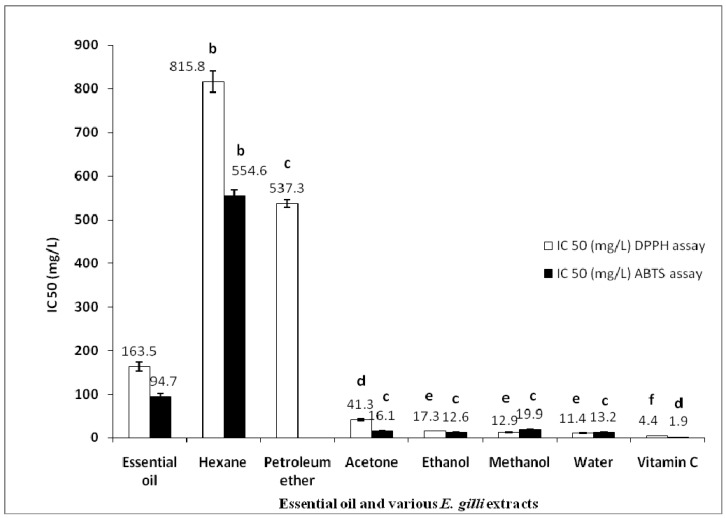
Free radical scavenging capacity [IC_50_ (mg/L)] of essential oil and *E. gillii* extracts. Petroleum ether extract: not analyzed by ABTS assay. Different letters (a–f) on the tops of the square columns were significantly different (*p* < 0.05).

Essential oil and different extracts were individually assessed for antioxidant activity using two tests: ABTS and DPPH free radical scavenging. Results are summarized in Figure 1.

#### 2.2.1. Essential Oil

The antioxidant activity of *E. gillii* essential oil was more important according to the ABTS assay, with an IC_50_ value of 94.7 ± 7.1 mg/L compared to the DPPH assay with an IC_50_ of 163.5 ± 10.7 mg/L (Figure 1). This activity is significant, especially since this essential oil are composed mainly of monoterpenes and sesquiterpene hydrocarbons and oxygenated ones which have a moderate activity compared to phenolics and vitamin C. Our findings revealed that the percentage of oxygenated monoterpenes was 53.6% and monoterpenes hydrocarbons was 12.2%. This result might be related to the antioxidant activity of our essential oil.

No antioxidant activity of *E. gillii* essential oil has been previously reported. According to literature, essential oils of *Eucalyptus* species native to south Tunisia (*E. gracilis*, *E. oleosa*, *E. salubris*, *E. salmonophloia*) seem to possess lower antioxidant properties than those of central Tunisia (*E. gillii*). In fact, Naceur *et al*. [11] have found that IC_50_ value of all essential oils were in the range 12.0–52.8 g/L, however, *E.* *gillii* essential oil showed an IC_50_ value 163.5 ± 10.7 mg/L according to our DPPH assay. The ABTS activity obtained for *E. gilii* essential oil (IC_50_ value of 94.7 ± 7.1 mg/L) was also more powerful than those described by Naceur *et al*. [11] for *E*. *salubris* (IC_50_ value of 273.2 ± 4.1 mg/mL).

Our results showed that *E.* *gillii* essential oil had a very good antioxidant activity compared to *E.* *radiata *[18] with a little antioxidant activity found in the ABTS assay (IC_50_= 484.3 ± 17.3 mg/L). As cited by Naceur *et al*. [19] the different parts of *E. oleosa* showed a small antioxidant activity (IC_50_ = 1,536.3 ± 40.5 mg/L) when using the DPPH test, and around 13 ± 0.6 mg/L when using the ABTS test.

#### 2.2.2. Various Extracts

For the DPPH assay (Figure 1), the water extract possessed the most important activity (IC_50_ = 11.4 ± 0.6 mg/L), followed by the methanol (IC_50_ = 12.9 ± 0.4 mg/L), ethanol (IC_50_ = 17.3 ± 0.4 mg/L), acetone (IC_50_ = 41.3 ± 2.1 mg/L), petroleum ether (IC_50_ = 537.3 ± 8.7 mg/L) and hexane (IC_50_ = 815.8 ± 24.8 mg/L) extracts. Ascorbic acid was used as positive control and exhibited an IC_50_ equal to 4.4 ± 0.2 mg/L.

With regards to the ABTS assay, the ethanolic extract presented good antioxidant activity (IC_50_ = 12.6 ± 0.7 mg/L), followed by the water (IC_50_ = 13.2 ± 1.2 mg/L), acetone (IC_50_ = 16.1 ± 0.5 mg/L), methanol (IC_50_ = 19.9 ± 0.2 mg/L) and hexane extracts (IC_50_ = 554.6 ± 13.3 mg/L). Ascorbic acid was used as positive control and exhibited an IC_50_ equal to 1.9 ± 0.1 mg/L.

Comparing the results of ABTS assay to those of the DPPH one, we can deduce that ABTS assay generally presents more activity.

According to the results found, the best antioxidant activities correspond to the polar fractions (water, ethanol, methanol). It is important to note that the species *E. gillii* has not been evaluated for antioxidant capacity. Hasegawa *et al*. [15] found that the high DPPH radical scavenging activity of phenolics isolated from *E. globulus* using hot water was about 3.8 µM (1.8 mg/L). The antioxidant activity of the methanol extract of *E. camaldulensis* using the DPPH assay was about an IC_50_ = 14.0 ± 0.2 mg/L. Extract was obtained by the extraction of 50 g of powdered *E. camaldulensis* leaves three times with 70% aqueous acetone as cited by Singab *et al*. [20]. The best IC_50 _value of our extracts is 11.4 ± 0.6 mg/L. This value is encouraging enough to prompt us to try to identify the molecules responsible for this activity.

Correlation between chemical composition of various *E. gillii* extracts and antioxidant activity was carried out for both the ABTS and DPPH assays. The composition of phenolics of various extracts seems to have a notable effect on the values of IC_50_ by the DPPH assay (R^2^ = 0.96) and IC_50_ by the ABTS assay (R^2^ = 0.71). Flavonoids were found to have also an effect on the variability of IC_50_ for ABTS assay (R^2^ = 0.70) and DPPH assay (R^2^ = 0.63), respectively. This correlation is not cited too often in the literature.

### 2.3. Antimicrobial Activity

#### 2.3.1. Minimum Inhibitory Concentration (MIC) of Essential Oil

The antimicrobial activity of *E. gillii* essential oil was evaluated by the determination of the Minimum Inhibitory Concentration (Table 4), which is the first concentration of added essential oil with which we do not notice visible microbial growth. According to results given in Table 4, we deduced that *E. gillii* essential oil exhibited a great potential against Gram positive bacteria, especially *Listeria monocytogenes* (MIC = 0.78 mg/mL). Concerning the essential oil activity against Gram negative bacteria, *Klebseilla pneumoniae *was the most sensitive (MIC = 2.34 mg/mL). We noticed also an important inhibition against yeast and fungi which was almost similar (3.12–3.90 mg/mL). To our knowledge, it is the first time that the antimicrobial activity of *E. gillii* was studied.

Several reports have studied the antimicrobial activity of *Eucalyptus* essential oils. Among these studies, *E. globulus *fruit essential oil exerted inhibitory activity against all Gram positive bacteria with MIC values between 0.06 and 1 mg/mL. For the tested Gram negative bacteria, *E. globulus* fruit essential oil did not show a substantial inhibition against *Pseudomonas aeruginosa*, *K. pneumoniae* and *Escherchia coli*. However it exhibited a moderate activity against yeasts with MIC values of 1–4 mg/mL. All antibiotic resistant bacteria were susceptible to *E. globulus* fruit essential oil with MIC values between 0.25 and 1 mg/mL, as cited by Mulyaningsih *et al*. [21]. It was also indicated that the MIC of *Eucalyptus* *dives* leaves essential oil from Austria against *Pseudomonas putida* was >0.8%, approximately 8 mg/L [6].

Other researchers have found that the antimicrobial activity of *E. globulus* essential oil (from Montenegro) was MIC = 0.9 mg/mL against *E. coli* and *Staphylococcus aureus*, MIC = 0.36 mg/mL against *Candida albicans*, MIC = 1.57 mg/mL against *P. aeruginosa* and *K. pneumoniae*. For *P. aeruginosa*, the MIC was about 3.13 mg/mL [22]. Comparing our results to these findings, we can deduce that *E. gillii* essential oil exhibited a good antimicrobial activity.

The relatively high antimicrobial activity was most likely due to the presence of compounds which have antimicrobial properties, particularly, 1,8-cineole which represents 43.8% of the *E.* *gillii* essential oil, and which is known to have relatively strong antimicrobial property against many important pathogens and spoilage organisms including *S. aureus*, *E. coli *and *B. subtilis *[23,24]. Compounds such as *p*-cymene, α-pinene and γ-terpinene also have relatively strong antimicrobial activity [25]. *E.* *gillii* essential oil was rich in oxygenated monoterpenes and this class comprised different active components which we suppose responsible for the antimicrobial power of our essential oil [26,27].

According to those results, we tried to explain the mechanism of the antimicrobial activity. This activity is related to an important characteristic of essential oil which is the hydrophobicity of their chemical components. This particularity allows them to be close to the lipid cell membrane of bacteria, disturbing the cellular structure and make it permeable; which can be responsible for the leakage of some ions and other metabolites, and eventually responsible for cellular death [27,28,29].

#### 2.3.2. Various Extracts

The antimicrobial activity of the different *E. gillii* extracts (methanol, ethanol, petroleum ether, acetone, hexane and water) was determined against three Gram positive and four Gram negative bacteria, two yeasts and three fungi. Results from the agar diffusion assay for antimicrobial activity of the various extracts are presented in Table 5. The inhibition zone, measured in millimeters, including the diameter of the disc, was used as the criterion for measuring the antimicrobial activity of *E. gillii* extracts. The inhibition zone diameters obtained were in the range of 11 to 20 mm. The methanol extract showed the highest activity against all microorganisms.

For Gram positive bacteria, the most sensitive microorganism was *B. subtilis*, followed by *S. aureus*, while *L. monocytogenes* was resistant to the hexane extract. Concerning Gram negative bacteria, *Salmonella enterica* was the most sensitive, with a 17 mm inhibition zone obtained with the petroleum ether extract, followed by *K. pneumoniae*, *P. aeruginosa* and *E. coli* which were more resistant against the various extracts. In the case of yeasts, *S. cervisiae* and *C. albicans* were both sensitive to all extracts, and the largest inhibition zone was 16 mm obtained with the methanol extract. Finally, for fungi, *Aspergillus parasiticus* was the most sensitive one, and all extracts exhibited a good activity, while *M. ramamniarus* and *Fusarium culmorum* were most resistant since we didn’t notice any inhibition zone with the acetone and petroleum ether extracts. The water extract didn’t exhibit antimicrobial activity against any of the strains. To conclude, the data proved that Gram positive bacteria were the most sensitive tested strains towards the various extracts. The MICs of different extracts have not been calculated because the samples only have moderate activity according to the disc diffusion assay method.

**Table 4:** Minimum inhibitory concentration for *E. gilii* essential oil.

	Minimum Inhibitory Concentration (mg/mL)												
	Gram positive bacteria				Gram negative bacteria				Yeast		Fungi		
Samples	*B. subtilis*		*S. aureus*	*L. monocytogenes*	*P. aeruginosa*	*S. enterica*	*E. coli*	*K. pneumoniae*	*S. cerevisiae*	*C. albicans*	*A. parasiticus*	*M. ramamnianus*	*F. culmorum*
Essential oil	3.90 ± 0.11 ^a^		3.12 ± 0.08^ b^	0.78 ± 0.02 ^c^	3.90 ± 0.09^ a^	3.12 ± 0.06^ b^	3.90 ± 0.14^ a^	2.34 ± 0.07^ d^	3.90 ± 0.10^ a^	3.12 ± 0.08^ b^	3.90 ± 0.12^ a^	3.12 ± 0.10^ b^	3.12 ± 0.08^ b^
Ampicillin	0.02 ± 0.00		0.02 ± 0.00	0.02 ± 0.00	0.01 ± 0.00	0.02 ± 0.00	0.02 ± 0.00	0.01 ± 0.00	0.02 ± 0.00	0.02 ± 0.00	0.02 ± 0.00	0.02 ± 0.00	0.02 ± 0.00
Nalidixic acid	0.02 ± 0.00		0.02 ± 0.00	0.02 ± 0.00	0.01 ± 0.00	0.02 ± 0.00	0.02 ± 0.00	0.01 ± 0.00	0.08 ± 0.00	0.02 ± 0.00	0.02 ± 0.00	0.02 ± 0.00	0.02 ± 0.00
Nystatin	0.02 ± 0.00		0.08 ± 0.00	0.08 ± 0.00	0.02 ± 0.00	0.02 ± 0.00	0.02 ± 0.00	0.02 ± 0.00	0.02 ± 0.00	0.02 ± 0.00	0.01 ± 0.00	0.02 ± 0.00	0.02 ± 0.00

Values within rows with uncommon superscripts (a–d) were significantly different (*p* < 0.05); ±: Standard deviation.

**Table 5:** Zone of inhibition of microorganisms by *E. gillii* extracts.

	Zones of inhibition (mm)											
	Gram positive bacteria			Gram negative bacteria				Yeast		Fungi		
Samples	*B. subtilis*	*S. aureus*	*L. monocytogenes*	*P. aeruginosa*	*S. enterica*	*E. coli*	*K. pneumoniae*	*S. cerevisiae*	*C. albicans*	*A. parasiticus*	*M. ramamnianus*	*F. culmorum*
Hexane	14 ± 0 ^a^	11 ± 0 ^a^	- ^a^	12 ± 0 ^a^	13 ± 0 ^a^	14 ± 0 ^a^	12 ± 0 ^a^	12 ± 0 ^a^	11 ± 0 ^a^	12 ± 0 ^a^	12 ± 0 ^a^	14 ± 0 ^a^
Petroleum ether	13 ± 0^a^	12 ± 0 ^a^	20 ± 0 ^b^	13 ± 0 ^a^	18 ± 0 ^b^	- ^b^	13 ± 0 ^a^	11 ± 0 ^a^	11 ± 0 ^a^	12 ± 0 ^a^	- ^b^	12 ± 0 ^a^
Acetone	16 ± 0^a^	13 ± 0 ^a^	13 ± 0 ^c^	13 ± 0 ^a^	14 ± 0 ^a^	12 ± 0 ^a^	12 ± 0 ^a^	14 ± 0 ^a^	15 ± 0 ^b^	11 ± 0 ^a^	- ^b^	- ^b^
Ethanol	16 ± 0 ^a^	18 ± 0 ^b^	11 ± 0 ^c^	15 ± 0^ b^	15 ± 0 ^a^	15 ± 0 ^a^	17 ± 0 ^b^	15 ± 0 ^b^	16 ± 0 ^b^	15 ± 0 ^b^	14 ± 0 ^a^	15 ± 0 ^c^
Methanol	20 ± 1 ^b^	15 ± 0 ^c^	16 ± 0 ^d^	- ^c^	13 ± 0 ^a^	17 ± 0 ^c^	13 ± 0 ^a^	17 ± 0 ^b^	13 ± 0 ^a^	13 ± 0 ^a^	13 ± 0 ^a^	16 ± 0 ^c^
Water	- ^c^	- ^d^	- ^a^	- ^c^	- ^c^	- ^b^	-^ c^	-^ c^	- ^c^	- ^c^	- ^b^	- ^b^
Ampicillin	54 ± 2 ^c^	20 ± 0 ^b^	31 ± 1 ^e^									
Nalidixic acid				19 ± 0 ^d^	27 ± 1 ^d^	30 ± 1 ^d^	28 ± 0 ^d^					
Nystatin								29 ± 1 ^d^	30 ± 1 ^d^	24 ± 0 ^d^	31 ± 1 ^c^	30 ± 1 ^d^

“–”: Absence of inhibition zone detected. Extract mass = 0.4 mg/disc. Antibiotic concentration = 0.33 mg/L. Values within columns with uncommon superscripts (a–d) were significantly different (*p* < 0.05); ±: Standard deviation.

Results prove that all tested extracts are endowed with an antimicrobial activity in spite of the morphological diversity of microorganism strains, concentration and variability of the chemical composition of every applied extract. Correlation between the chemical composition of each family and the antimicrobial activity of each strain were evaluated for the disc diffusion assay. The results show that anthocyanins seem to have a major effect on the growth of *B. subtilis* (R^2^ = 0.79): the size of the inhibition zone increases when the amount of anthocyanins also increases.

## 3. Experimental

### 3.1. Chemicals

All chemicals used were of analytical reagent grade. All reagents were purchased from Sigma-Aldrich-Fluka (Saint-Quentin, France).

### 3.2. Collection of Plant Material

*E. gillii *leaves were picked on April 2009 from trees growing in the Hajeb Layoun arboretum, located in Kairouan governorship in Tunisia. They were stored at a dry place for fifteen days. Specimens were identified at the Regional Station of the National Institute of Research in Farming Studies, Waters and Forests. A voucher specimen (reference 0109) was deposited at the Department of Biology. The arboretum was established in April 1960 and the plant was originally imported from Austria. Dried leaves were subjected to hydrodistillation and preparation of the various extracts.

### 3.3. Extraction

#### 3.3.1. Isolation of Essential oil

One hundred g of dried leaves were crushed (2.5 mm of diameter), then subjected to hydrodistillation (500 mL water) in a Clevenger type apparatus for 3 h. The essential oil obtained was dried with anhydrous sodium sulfate and kept in amber vials at 4 °C for further analysis. Na_2_SO_4_ was removed before use of the essential oil. The extraction yield was calculated using the following formula: yield = (V_EO_ × 100)/D.M (D.M: dry material; V_EO_: volume of essential oil).

#### 3.3.2. Preparation of Extracts

The extraction method was sequential extraction with solvents of increasing polarity. Solvents used were: hexane, petroleum ether, acetone, ethanol, methanol and water. Ten g of harvested leaves, finely crushed, were placed in hexane (100 mL) for 16 h under frequent agitation at ambient pressure and temperature. The mixture was filtered using Wattman paper (GF/A, 110 mm). The solvent was evaporated using a rotary evaporator under vacuum at 35 °C. Then, the firstly extracted powder was extracted with petroleum ether under the same conditions as with hexane. The same procedure was applied for the following solvents. Extracts were kept in amber vials and stored at 4 °C for further analysis. 

### 3.4. Gas Chromatography and Gas Chromatography-Mass Spectrometry

Quantitative and qualitative analysis of the essential oil was carried out by gas chromatography-flame ionization detection (GC-FID) and gas chromatography-mass spectrometry (GC-MS). Gas chromatography analyses were carried out on a Varian Star 3400 (Les Ulis, France) Cx chromatograph fitted with a fused silica capillary DB-5MS column (5% phenylmethylpolysiloxane, 30 m × 0.25 mm, film thickness 0.25 µm). Chromatographic conditions were 60 °C to 260 °C temperature rise with a gradient of 5 °C/min and 15 min isotherm at 260 °C. A second gradient was applied to 340 °C at 40 °C/min. Total analysis time was 57 min.

For analysis, essential oil was dissolved in petroleum ether. One microliter of sample was injected in 1:10 split mode. Helium (purity 99.999%) was used as carrier gas at 1 mL/min. The injector was operated at 200 °C. The mass spectrometer (Varian Saturn GC/MS/MS 4D) was adjusted for an emission current of 10 µA and electron multiplier voltage between 1,400 and 1,500 V. Trap temperature was 220 °C and that of the transfer line was 250 °C. Mass scanning was from 40 to 650 amu.

Compounds were identified by comparison of their KI (retention indices) relative to C_5_-C_24_ *n*-alkanes obtained on a nonpolar DB-5MS column, with those provided in the literature, by comparison of their mass spectra with those recorded in NIST 08 (National Institute of Standards and Technology) and reported in published articles and by co-injection of available reference compounds [α-pinene (98%, Aldrich); *p*-cymene (99%, Aldrich); limonene (≥99.0%, Fluka); 1,8-cineole (99%, Aldrich); γ-terpinene (97%, Aldrich); α-terpinolene (≥95.0%, Aldrich); fenchol (≥99.0%, Fluka); borneol (97%, Aldrich); myrtenal (98%, Aldrich); myrtenol (≥95.0%, Aldrich); verbenone (94%, Aldrich); pulegone (≥98.5%, Fluka); cuminaldehyde (98%, Aldrich); *p*-cymen-7-ol (97%, Aldrich); globulol (≥98.5%, Aldrich); guaiol (97%, Aldrich); β-eudesmol (>90%, Sigma)]. The samples were analyzed in duplicate. The percentage composition of the essential oil was computed by the normalization method from the GC peak areas, assuming identical mass response factor for all compounds. Results were calculated as mean values of three injections from essential oil, without using correction factors. All determinations were performed in triplicate and averaged.

### 3.5. Free Radical Scavenging Activity: DPPH Test

Antioxidant scavenging activity was studied using 1,1-diphenyl-2-picrylhydrazyl free radical (DPPH) as described by Blois [30] with some modifications. Various dilutions of samples (extracts or essential oil, 1.5 mL) were mixed with 0.2 mM methanolic DPPH solution (1.5 mL). After an incubation period of 30 min at 25 °C, the absorbance at 520 nm was measured. The wavelength of maximum absorbance of DPPH, was recorded as A_(sample)_, using a spectrophotometer (Helios, Unicam, Cambridge, UK). A blank experiment was also carried out applying the same procedure to a solution without the test material and the absorbance was recorded as A_(blank)_. The free radical-scavenging activity of each solution was then calculated as percent inhibition according to the following equation: % inhibition = ((A_(blank)_ − A_(sample)_)/A_(blank)_) × 100.

Antioxidant activity of standard or samples was expressed as IC_50_, defined as the concentration of the test material required to cause a 50% decrease in initial DPPH concentration. Ascorbic acid was used as a standard. All measurements were performed in triplicate.

### 3.6. ABTS Radical-Scavenging Assay

The radical scavenging capacity of antioxidants for the ABTS (2,2'-azinobis-3-ethylbenzothiazoline-6-sulphonate) radical cation was determined as follows: ABTS was generated by mixing a 7 mM solution of ABTS at pH 7.4 (5 mM NaH_2_PO_4_, 5 mM Na_2_HPO_4_ and 154 mM NaCl) with 2.5 mM potassium persulfate (final concentration) followed by storage in the dark at room temperature for 16 h before use. The mixture was diluted with persulfate buffer to give an absorbance of 0.70 ± 0.02 units at 734 nm using a spectrophotometer (Helios, Unicam, Cambridge, UK). For each sample, diluted solution of the essential oil (100 μL) was allowed to react with fresh ABTS solution (900 μL), and then the absorbance was measured 6 min after initial mixing. Ascorbic acid was used as a standard. The capacity of free radical scavenging was expressed by IC_50_ (mg/L) values which represents the concentration required to scavenge 50% of ABTS radicals. The capacity of free radical scavenging IC_50_ was determined using the same equation previously used for the DPPH method. All measurements were performed in triplicate.

### 3.7. Total Amount of Phenolic Compounds

The total phenolics of each extract were determined by the Folin-Ciocalteu [31] method. Preparation of 2 N Folin and Ciocalteu reagent (Fluka): dissolve sodium tungstate (10 g) and sodium molybdate (2.5 g) in water (70 mL). Add 85% phosphoric acid (5 mL) and concentrated hydrochloric acid (10 mL). Reflux for 10 h. Add lithium sulfate (15 g), water (5 mL), and 1 drop of bromine. Reflux for 15 min. Cool to room temperature and bring to 100 mL with water. Hexavalent phosphomolybdic/phosphotungstic acid complexes are formed in solution.

The diluted aqueous solution of each extract (0.1 mL) was mixed with Folin-Ciocalteu reagent (0.5 mL at 0.2 N). This mixture was allowed to stand at room temperature for 5 min and then sodium carbonate solution (0.4 mL at 75 g/L) was added. After 1 h of incubation, the absorbances were measured at 765 nm against water blank using a Helios spectrophotometer (Unicam, Cambridge, UK). A standard calibration curve was plotted using gallic acid (0 to 200 mg/L). The results were expressed as mg of gallic acid equivalents (GAE)/1 g of dry mass.

### 3.8. Condensed Tannin Content

Catechins and proanthocyanidins reactive to vanillin were analyzed by the vanillin method [32,33]. One milliliter (0.5 mL) of each extract solution was placed in a test tube and 2 mL of vanillin (1% in 7 M H_2_SO_4_) in an ice bath and then incubated at 25 °C. After 15 min, the absorbance of the solution was read at 500 nm. Concentrations were calculated as gram catechin equivalents (CE)/kg dry mass from a calibration curve.

### 3.9. Total Flavonoids Determination

The total flavonoids were estimated according to the Dowd method as adapted by Arvouet-Grand *et al.* [34]. A diluted methanolic solution (0.5 mL) of each extract was mixed with a solution (0.5 mL) of aluminum trichloride (AlCl_3_) in methanol (2%). The absorbance was read at 415 nm after 15 min against a blank sample consisting of a methanol (0.5 mL) and extract (0.5 mL) without AlCl_3_. Quercetin was used as reference compound to produce the standard curve, and the results were expressed as gram of quercetin equivalents (QE)/kg of dry mass.

### 3.10. Determination of Total Anthocyanin Content

Total anthocyanin content was measured with the pH differential absorbance method, as described by Cheng *et al*. [35]. Briefly, absorbance of the extract was measured at 510 and 700 nm in buffers at pH 1 (hydrochloric acid-potassium chloride, 0.2 M) and 4.5 (acetic acid-sodium acetate, 1 M). The wavelength reading was performed after 15 min of incubation. Anthocyanin content was calculated using a molar extinction coefficient (*ε*) of 29,600 (cyanidin-3-glucoside) and absorbance of A = [(A_510_− A_700_) pH 1.0 − (A_510_− A_700_) pH4.5]. Results were expressed as milligram cyanidin-3-glucoside equivalent (C3GE)/kg of dry mass.

### 3.11. Antimicrobial Activity

#### 3.11.1. Microbial Strains

All strains were obtained from the Laboratory of Chemical Engineering, Bioprocess Systems Microbiens Department, Ecole Nationale Supérieure Agronomique de Toulouse. The essential oil was individually tested against a panel of microorganisms. Seven bacteria including three Gram positive (*Staphylococcus aureus* CIP 7625, *Listeria monocytogenes *Scott A 724, *Bacillus subtilis* ATCC 6633), and four Gram negative (*Pseudomonas aeruginosa CIPA22*, *Salmonella enterica CIP833*, *Escherchia coli* ATCC 10536, *Klebseilla pneumoniae* CIP 8291) were used. Two yeasts (*Saccharomyces cerevisiae* EDV 436 and *Candida albicans* IPA 200) and three fungi (*Aspergillus parasiticus* NRRL 3174, *Mucor ramamnianus *NRLL 1829 and *Fusarium culmorum *NRRL 3288) were also tested. The bacterial and yeast strains were cultured on nutrient agar for 48 h at 37 °C, while fungi were propagated in PDA (*F. culmorum*) and in ISPD_2_ (*A. parasiticus*, *M. ramamnianus*) at 30 °C for 48 h to 3 days before used. All microorganisms were stocked at −6 °C in appropriate conditions and were regenerated twice before use in the manipulations.

Growing stock culture was normalized through various cycles of growth. Indeed, the density varies between bacteria, yeasts and fungi. The bacterial suspensions were prepared from pre-cultures of 24 h on Trypticase-soy at 37 °C in sterile distilled water, and adjusted to 4 × 10^6^ bacteria/mL. Spores of strains fungi are recovered using a swab to surface of a culture of seven days on sabouraud agar and suspended in sterile distilled water containing 0.05% polysorbate 80 to a better dispersion of spores. After vortex treatment, the spore suspension is adjusted to the desired concentration by cell count in Thomas cells by light microscopy (×400). The concentration of spores suspensions (fungi and yeasts) was about 25 × 10^5^ cells per mL.

The choice of the used concentrations of inoculums size is traduced by the influence of inoculum size on antimicrobial activity. In fact, a bacterial population introduced into a new culture medium has a fairly characteristic change. The concentration of chosen inoculums must be in the exponential phase to ensure an optimal response of microorganisms to various antimicrobial agents.

#### 3.11.2. MIC Agar Dilution Assay

The minimum inhibitory concentration (MIC) values of microorganisms were studied, based on the agar dilution method. The essential oils of *E. gillii *were dissolved with methanol (400 μL of essential oil in 400 μL methanol). The essential oil dissolved was added aseptically to sterile nutrient agar supplemented with Tween 80 (0.5%) at appropriate volume to produce the concentration range of 0.5–20 mg/mL. No antimicrobial activity noticed for methanol.

The resulting nutrient agar solutions were immediately poured into Petri dish after vortexing and allowed to solidify. The dish was left to cool down and to solidify at room temperature for 30 min. The plates were spotted, then inoculated with 1 μL of bacterial strains (4 × 10^6^ cells/mL), of yeast and fungi (25 × 10^5^ spores/mL). Tests were carried out in duplicate.

Ampicillin and nalidixic acid (0.5–20 mg/L) were used as positive reference standards to determine the sensitivity of Gram positive and Gram negative bacterial species tested respectively. Nystatin (0.5–20 mg/L) was used as a positive reference standard to determine the sensitivity of fungi and yeast species. These plates, after staying at 4 °C for 2 h, were incubated at 37 °C for bacteria and at 30 °C for 48 h for yeast and fungi. Tests were carried out in duplicate.

#### 3.11.3. Disc-Diffusion Assay

The paper disc diffusion method was employed for the determination of antimicrobial activity of various *E. gillii* extracts [36]. Briefly, a suspension of the tested microorganism (0.1 mL of 10^8^ cells per mL for bacteria and 25 × 10^5^ cells per mL for fungi) was spread on nutrient agar. The discs have a diameter 9 mm, and 40 µL of the diluted extract in methanol (1/100 from the initial concentration) were injected and placed on the inoculated plates. Ampicillin and nalidixic acid (40 µg/disc) were used as positive reference standards to determine the sensitivity of Gram positive, Gram negative bacterial species tested, respectively. Nystatin (40 µg/disc) was used as positive reference standard to determine the sensitivity of fungus and yeasts species. No antimicrobial activity was noticed for methanol.

These plates, after staying at 4 °C for 2 h, were incubated for 48 h at 37 °C for bacteria and at 30 °C for yeasts and fungus. Antimicrobial activity was evaluated by measuring the zone of inhibition against the test organism. The diameter of the inhibition zones were measured in millimeters. Tests were carried out in triplicate.

The sensitivity to the individual extracts was classified by the diameter of the inhibition zones as indicated by Moreira *et al.* [37] with a small modification. Not sensitive for total diameter smaller than 9 mm; sensitive for total diameter 10–15 mm; Very sensitive for total diameter 16–20 mm; extremely sensitive for total diameter larger than 20 mm.

### 3.12. Statistical Analysis

All data were expressed as means ± standard deviations of triplicate measurements. The confidence limits were set at *p* < 0.05. Standard deviations (SD) did not exceed 5% for the majority of the values obtained. Correlation coefficients (R^2^) to determine the relationship between chemical composition and antioxidant or biological activity were calculated.

## 4. Conclusions

*Eucalyptus* essential oil and its extracts are known for their therapeutic virtue owing to their antimicrobial activity. They may be used in food industry, not only as flavoring agents but also as preservatives [5,38]. In this work, the major constituents of *E. gillii* leaves essential oil were 1,8-Cineole (43.8%), *p*-cymene (14.2%), α-pinene (10%). High antioxidant activity was detected (ethanol extract, IC_50_ = 12.6 ± 0.7 mg/L) by the ABTS assay. Further work is in progress to purify the extract that gave a good antioxidant activity to identify the molecule(s) responsible for this activity. The results of antimicrobial activity showed that *L. monocytogenes* seems to be the most sensitive Gram positive bacteria, (MIC = 0.78 mg/mL); *K. pneumonia* presents the lowest MIC (2.34 mg/mL) among the other Gram negative bacteria. Yeast and fungi present an approximate MIC of almost 3.12 to 3.9 mg/mL. These research findings lead us to conclude that *E. gillii* essential oil and various extracts, mainly the methanolic one, could be considered as potential alternatives for synthetic bactericides and natural antioxidants for use in the food industry along with their possible applications in the pharmaceutical industry for the prevention or treatment of pathogenesis caused by microorganisms and free radicals.

## Supplementary Materials

Supplementary File 1

## References

[1] Lambert R.J.W., Skandamis P.N., Coote P., Nychas G.J.E. (2001). A study of the minimum inhibitory concentration and mode of action of oregano essential oil, thymol and carvacrol. J. Appl. Microbiol..

[2] Guidance for Industry: Frequently Asked Questions about GRAS. http://www.fda.gov/Food/GuidanceComplianceRegulatoryInformation/GuidanceDocuments/FoodIngredientsandPackaging/ucm061846.htm/.

[3] Pittman C.I., Pendleton S., Bisha B., O’Bryan C.A., Belk K.E., Goodridge L., Crandall P.G., Ricke S.C. (2011). Activity of citrus essential oils against *Escherichia coli* O157:H7 and *Salmonella* spp. and effects on beef subprimal cuts under refrigeration. J. Food Sci..

[4] Karabagias I., Badeka A., Kontominas M.G. (2011). Shelf life extension of lamb meat using thyme or oregano essential oils and modified atmosphere packaging. Meat Sci..

[5] Djenane D., Aïder M., Yangüela J., Idir L., Gómez D., Roncalés P. (2012). Antioxidant and antibacterial effects of Lavandula and Mentha essential oils in minced beef inoculated with *E*. *coli* O157:H7 and *S*. *aureus* during storage at abuse refrigeration temperature. Meat Sci..

[6] Oussalah M., Caillet S., Saucier L., Lacroix M. (2006). Antimicrobial effects of selected plant essential oils on the growth of a *Pseudomonas putida* strain isolated from meat. Meat Sci..

[7] Brooke M.I.H., Kleinig D.A. (2004). Field Guide to Eucalypts.

[8] Guimarães R., João Sousa M., Ferreira I.C.F.R. (2010). Contribution of essential oils and phenolics to the antioxidant properties of aromatic plants. Ind. Crops Prod..

[9] Jaimand K., Assareh M.H., Rezaee M.B. (2006). Volatile oil constituents of leaves of the *Eucalyptus gillii *maiden and *E. microcarpa *subsp. *macrocarpa *Hook from Iran. Iran. J. Pharm. Res..

[10] Giamakis A., Kretsi O., Chinou I., Spyropoulos C.G. (2001). *Eucalyptus camaldulensis*: Volatiles from immature flowers and high production of 1,8-cineole and *â*-pinene by *in vitro *cultures. Phytochemistry.

[11] Naceur H., Romdhane M., Lebrihi A., Mathieu F., Couderc F., Abderraba M., Khouja M.L., Bouajila J. (2010). *Eucalyptus *(*gracilis*, *oleosa*, *salubris*, and * salmonophloia*) essential oils: Their chemical composition and antioxidant and antimicrobial activities. J. Med. Food.

[12] Amakuraa Y., Uminoa Y., Tsujia S., Itob H., Hatanob T., Yoshidab T., Tonogaia Y. (2002). Constituents and their antioxidative effects in *Eucalyptus* leaf extract used as a natural food additive. Food Chem..

[13] Li H., Madden J.L., Pottis B.M. (1997). Variation in leaf waxes of the Tasmanian *Eucalyptus* Species-I *Subgenus Symphomyrtus*. Biochem. Syst. Ecol..

[14] Rahman A., Talukder F.A. (2006). Bioefficacy of some plant derivatives that protect grain against the pulse beetle, *Callosobruchus maculatus*. J. Insect Sci..

[15] Hasegawa T., Takano F., Takata T., Niiyama M., Oyha T. (2008). Bioactive monopterpene glycosides conjugated with gallic acid from the leaves of *Eucalyptus globules*. Phytochemistry.

[16] Ennajar M., Bouajila J., Lebrihi A., Mathieu F., Abderraba M., Raies A., Romdhane M.  (2009). Chemical composition and antimicrobial and antioxidant activities of essential oils and various extracts of *Juniperus phoenicea* L. (Cupressacees). J. Food Sci..

[17] Chapuis-Lardy L., Contour-Ansel D., Bernhard-Reversat F. (2002). High performance liquid chromatography of water, soluble phenolics in leaf litter of three Eucalyptus *Hybrids* (Congo). Plant Sci..

[18] Bendaoud H., Bouajila J., Rhouma A., Savagna A., Romdhane M. (2009). GC/MS analysis and antimicrobial and antioxidant activities of essential oil of *Eucalyptus radiate*. J. Sci. Food Agric..

[19] Naceur Ben Marzoug H., Romdhane M., Lebrihi A., Mathieu F., Couderc F., Abderraba M., Khouja M.L., Bouajila J. (2011). *Eucalyptus oleosa* essential oils: Chemical composition and antimicrobial and antioxidant activities of the oils from different plant parts (stems, leaves, flowers and fruits. Molecules.

[20] Singab A.N., Ayoub N., Al Sayed E., Martiskainen O., Sinkkonen J., Pihlaja K. (2011). Phenolic constituents of *Eucalyptus camaldulensis* Dehnh, with potential antioxidant and cytotoxic activities. Rec. Nat. Prod..

[21] Mulyaningsih S., Sporer F., Zimmermann S., Reichling J., Wink M. (2010). Synergistic properties of the terpenoids aromadendrene and 1-8cineole from the essential oil of *Eucalyptus globules* against antibiotic susceptible and antibiotic resistant pathogens. Phytomedecine.

[22] Damjanovic-Vratnica B., Dakov T., Sukovic D., Damjanovic J. (2011). Antimicrobial effect of essential oil isolated from *Eucalyptus globules Labill* from Montenegro. Czech J. Food Sci..

[23] Rosato A., Vitali C., de Laurentis N., Armenise D., Milillo M. (2007). Antibacterial effect of some essential oils administered alone or in combination with Norfloxacin. Phytomedicine.

[24] Sonboli A., Babakhani B., Mehrabian A.R. (2006). Antimicrobial activity of six constituents of essential oil from Salvia. Z. Naturforsch. C.

[25] Bakkali F., Averbeck S., Averbeck D., Idaomar M. (2008). Biological effects of essential oils—A review. Food Chem. Toxicol..

[26] Juven B.J., Kanner J., Schved F., Weisslowicz H. (1994). Factors that interact with the antibacterial action of thyme essential oil and its active constituents. J. Appl. Bacteriol..

[27] Ultee A., Slump R.A., Steging G., Smid E.J. (2000). Antimicrobial activity of carvacrol toward *Bacillus cereus* on rice. J. Food Protec..

[28] Sikkema J., de Bont J.A.M., Poolman B. (1994). Interactions of cyclic hydrocarbons with biological membranes. J. Biolog. Chem..

[29] Burt S. (2004). Essential oils: Their antibacterial properties and potential applications in foods—A review. Int. J. Food Microbiol..

[30] Blois M.S. (1958). Antioxidant determinations by the use of a stable free radical. Nature.

[31] Folin O., Ciocalteu V. (1927). On tyrosine and tryptophane determination in proteins. J. Biol. Chem..

[32] Broadhurst R.B., Jones W.T. (1978). Analysis of condensed tannins using acidified vanillin. J. Sci. Food Agric..

[33] Naczk M., Zadernowski R., Shahidi F. (2001). Protein precipitating capacity of condensed tanins of beach pea, canola hulls, evening primrose and faba bean. J. Agric. Food Chem..

[34] Arvouet-Grand A., Vennat B., Pourrat A., Legret P. (1994). Standardisation d’un extrait de propolis et identification des principaux constituants. J. Pharm. Belg..

[35] Cheng G.W., Breen P. (1991). Activity of phenylalanine ammonialyase (PAL) and concentrations of anthocyanins and phenolics in developing strawberry fruit. J. Am. Soc. Hort. Sci..

[36] (1999). Methods for Determining Bactericidal Activity of antimIcrobial Agents: Approved Guideline.

[37] Moreira M.R., Ponce A.G., Del Valle C.E., Roura S.I. (2005). Inhibitory parameters of essential oils to reduce a food borne pathogen. LWT Food Sci. Technol..

[38] Djenane D., Yangüela J., Amrouche T., Boubrit S., Boussad N., Roncalés P. (2011). Chemical composition and antimicrobial effects of essential oils of *Eucalyptus globulus*, *Myrtus communis* and *Satureja hortensis* against *Escherichia coli* O157:H7 and *Staphylococcus aureus* in minced beef. Food Sci. Technol. Int..

