# Supplementary material for: Chemical Composition and in Vitro Evaluation of the Antioxidant and Antimicrobial Activities of Eucalyptus gillii Essential Oil and Extracts
Source: Molecules. 2012 Aug 9;17(8):9540–58. doi: 10.3390/molecules17089540 (PMC6268698; doi:10.3390/molecules17089540)
Supplement: Supplementary File 1 [file molecules-17-09540-s001.pdf]

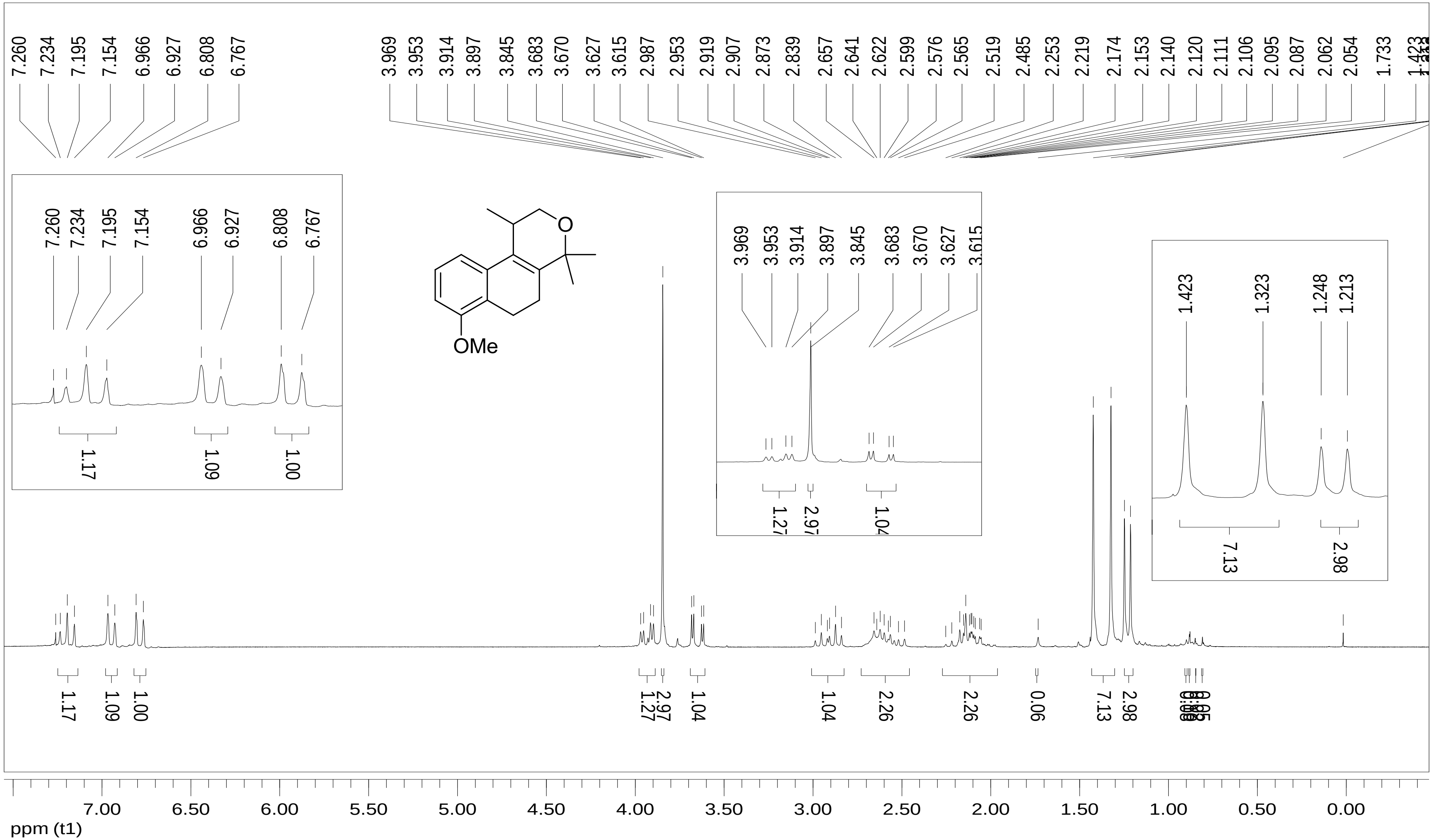

^{1}H-NMR (200 MHz, $CDCl_3$) spectrum of **2a**

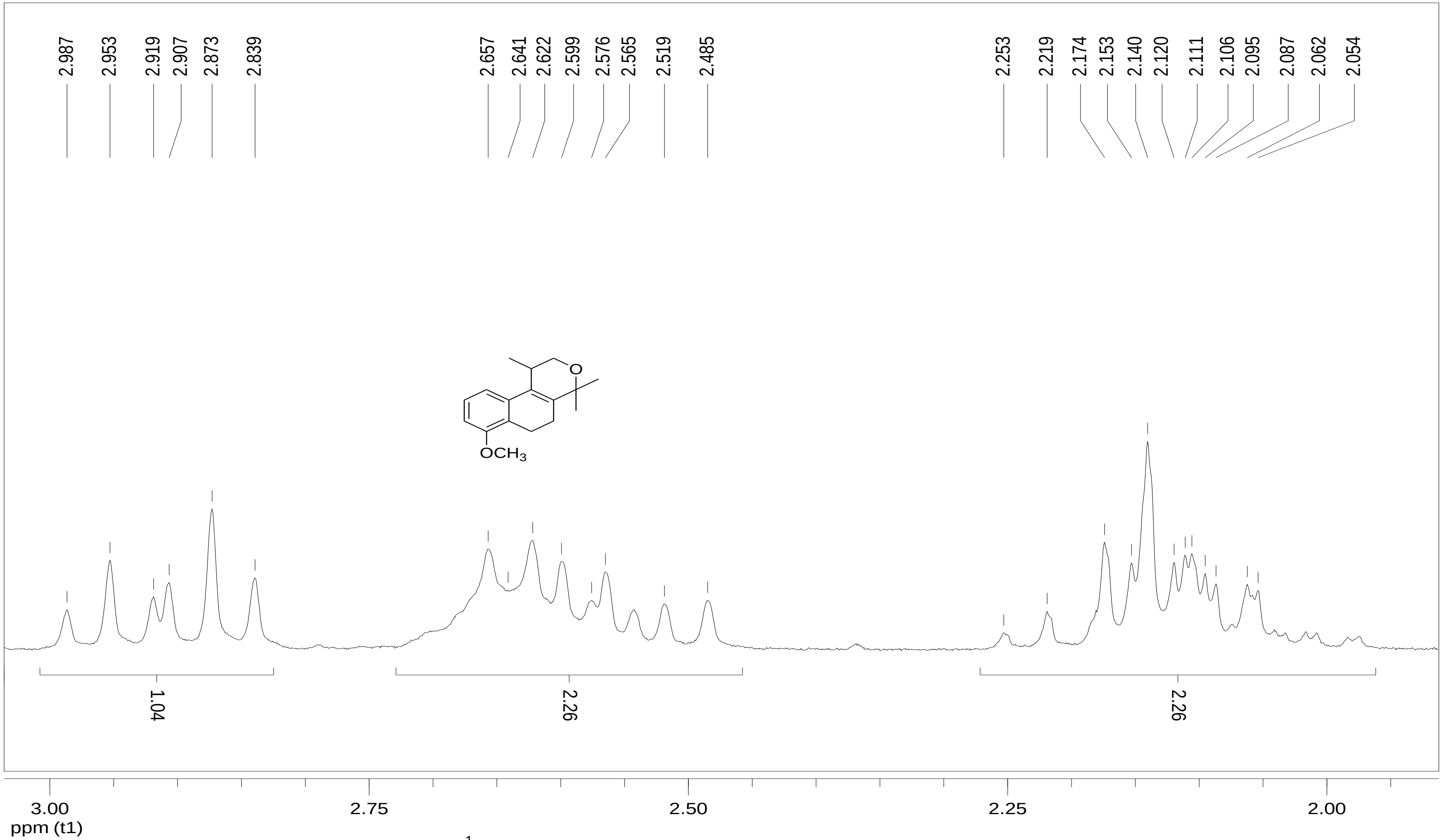

^{1}H-NMR (200 MHz, $CDCl_3$) selected expansions of **2a**

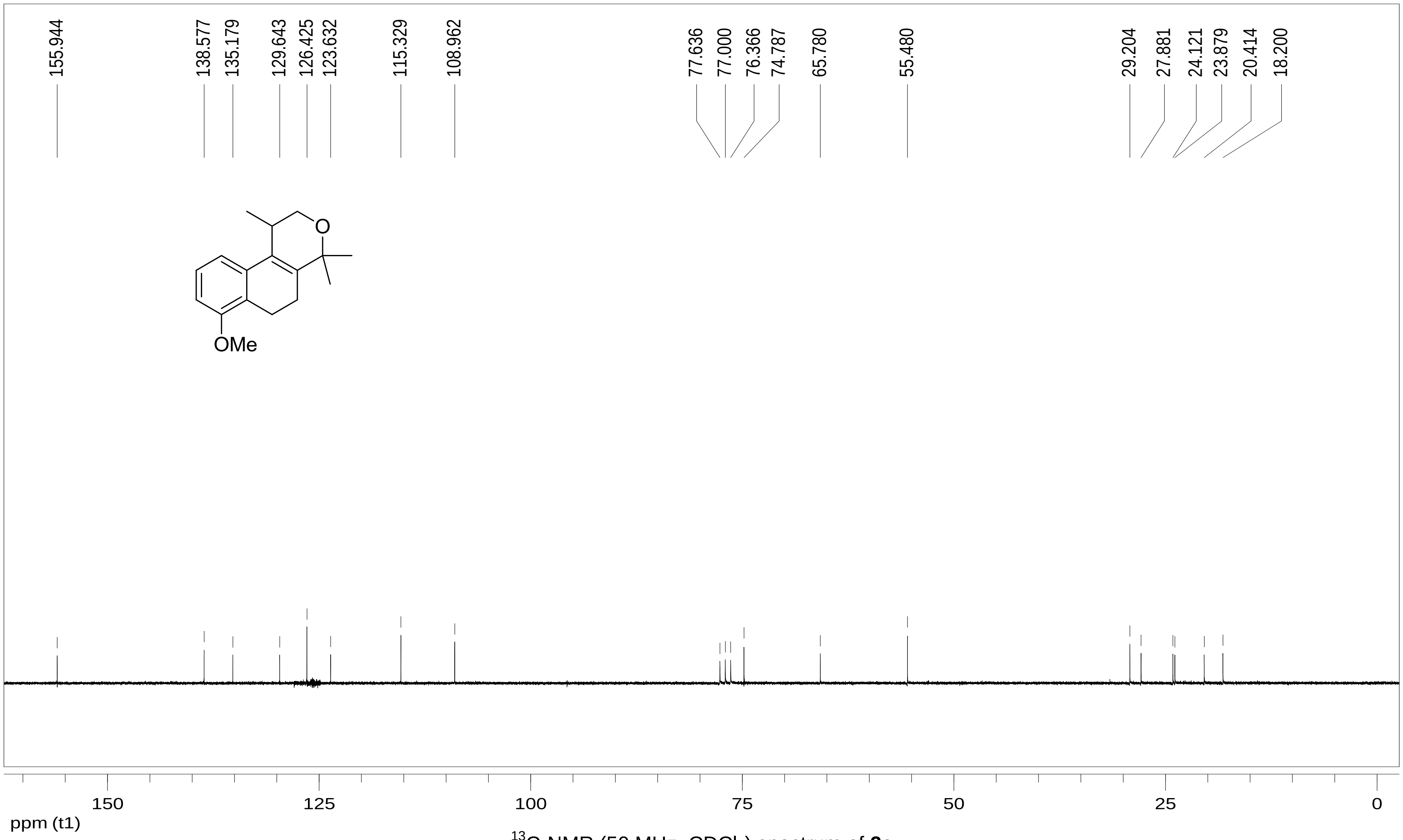

^{13}C-NMR (50 MHz, $CDCl_3$) spectrum of **2a**

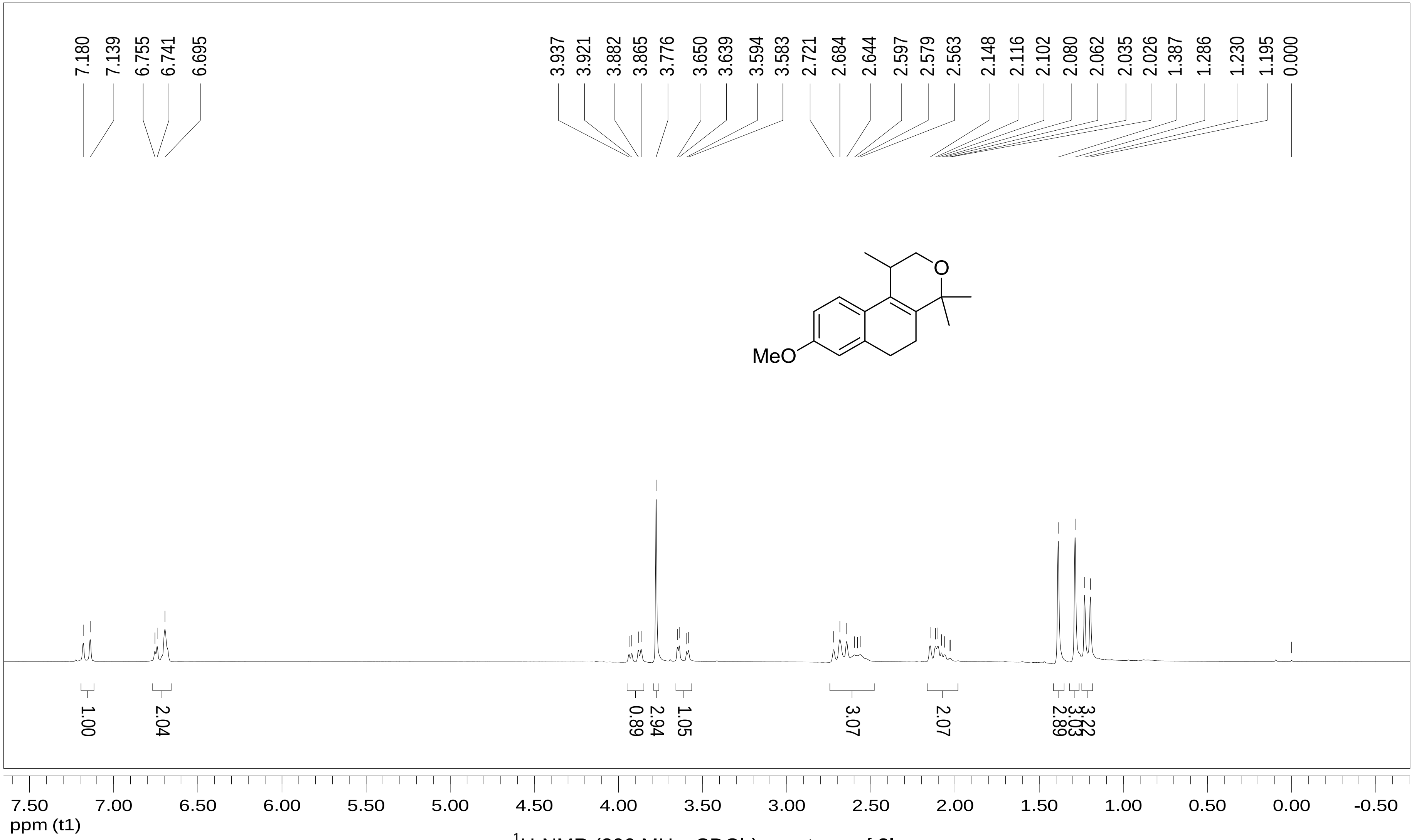

^{1}H-NMR (200 MHz, $CDCl_3$) spectrum of **2b**

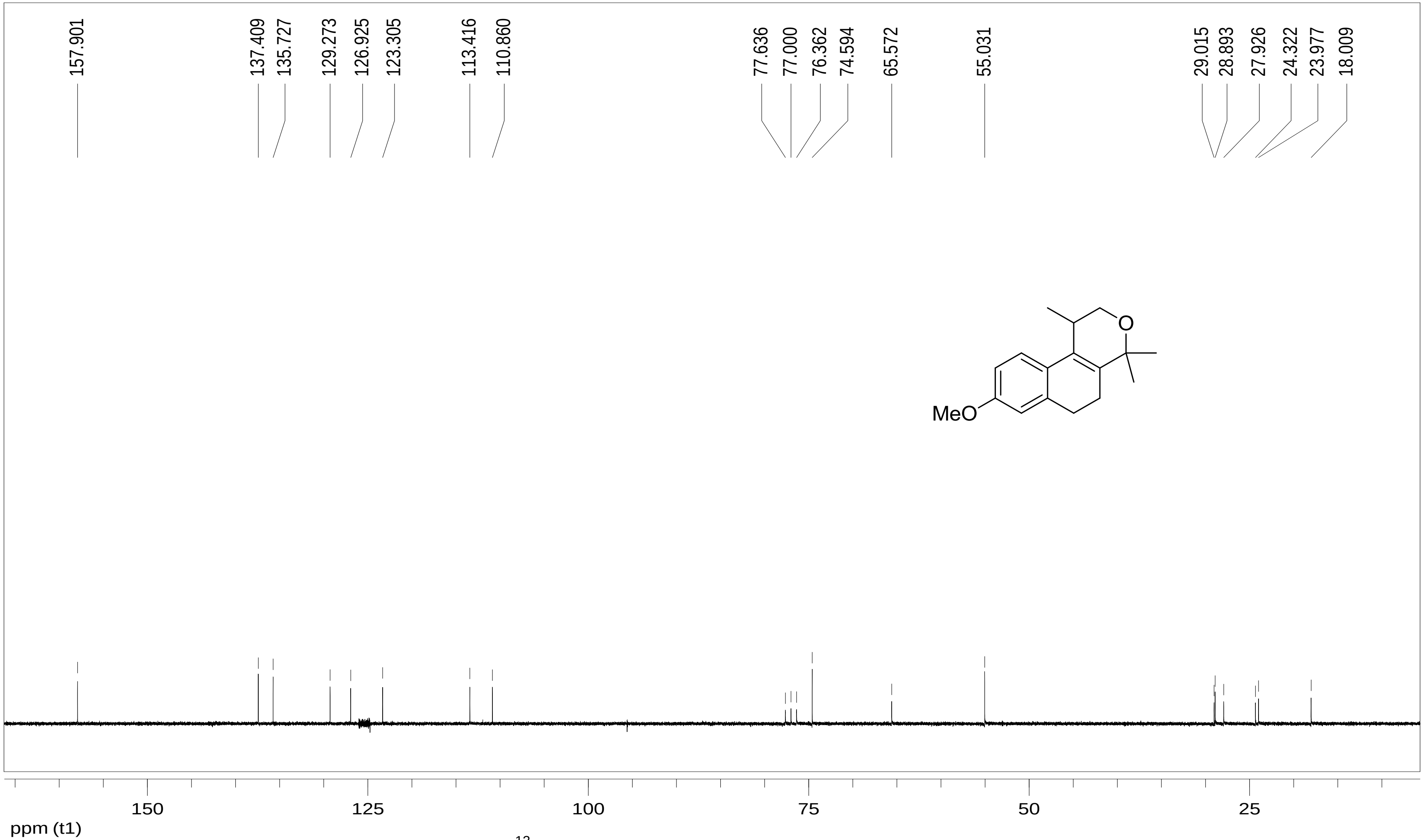

^{13}C-NMR (50 MHz, $CDCl_3$) spectrum of **2b**

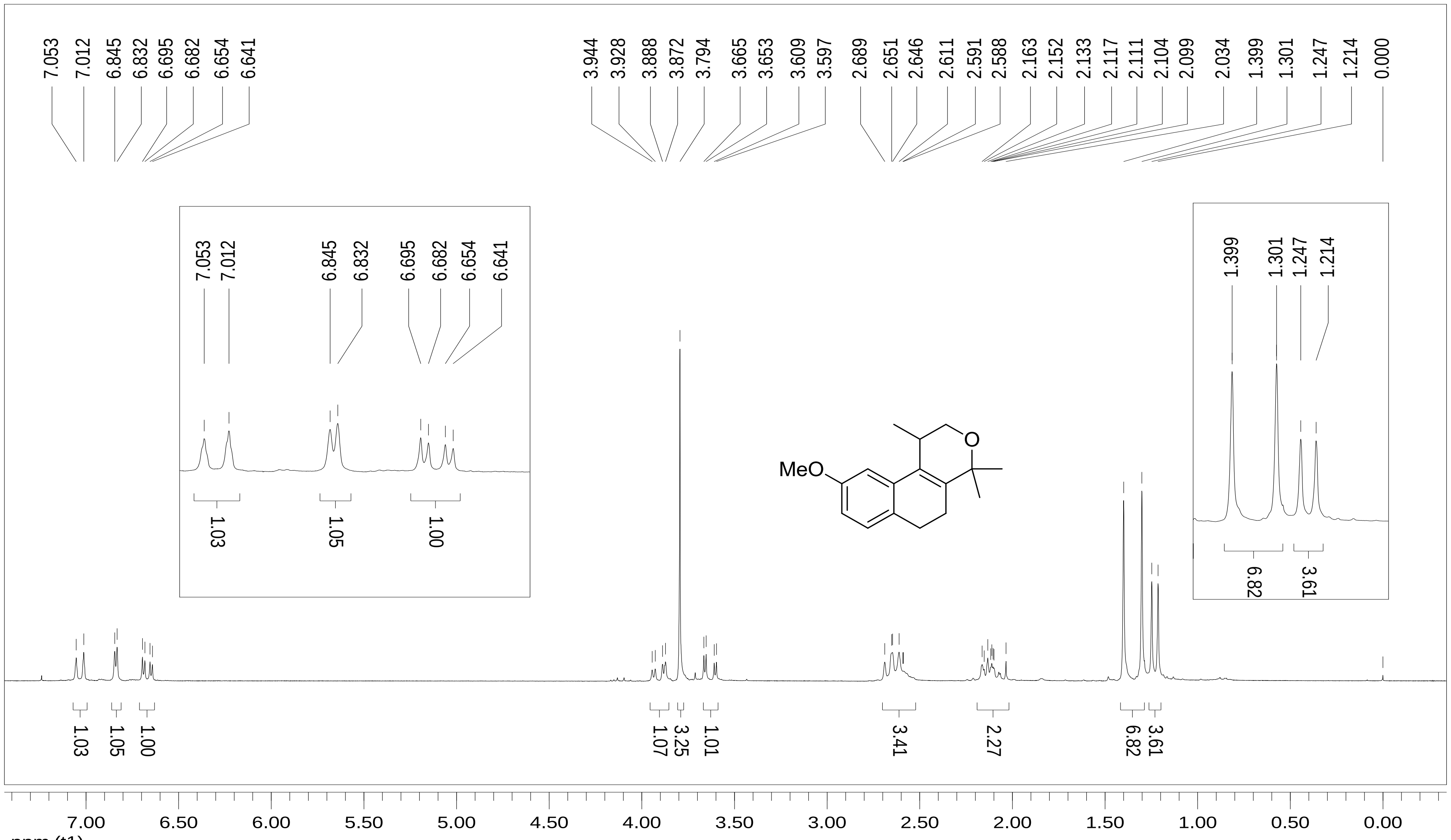

^{1}H-NMR (200 MHz, $CDCl_3$) spectrum of **2c**

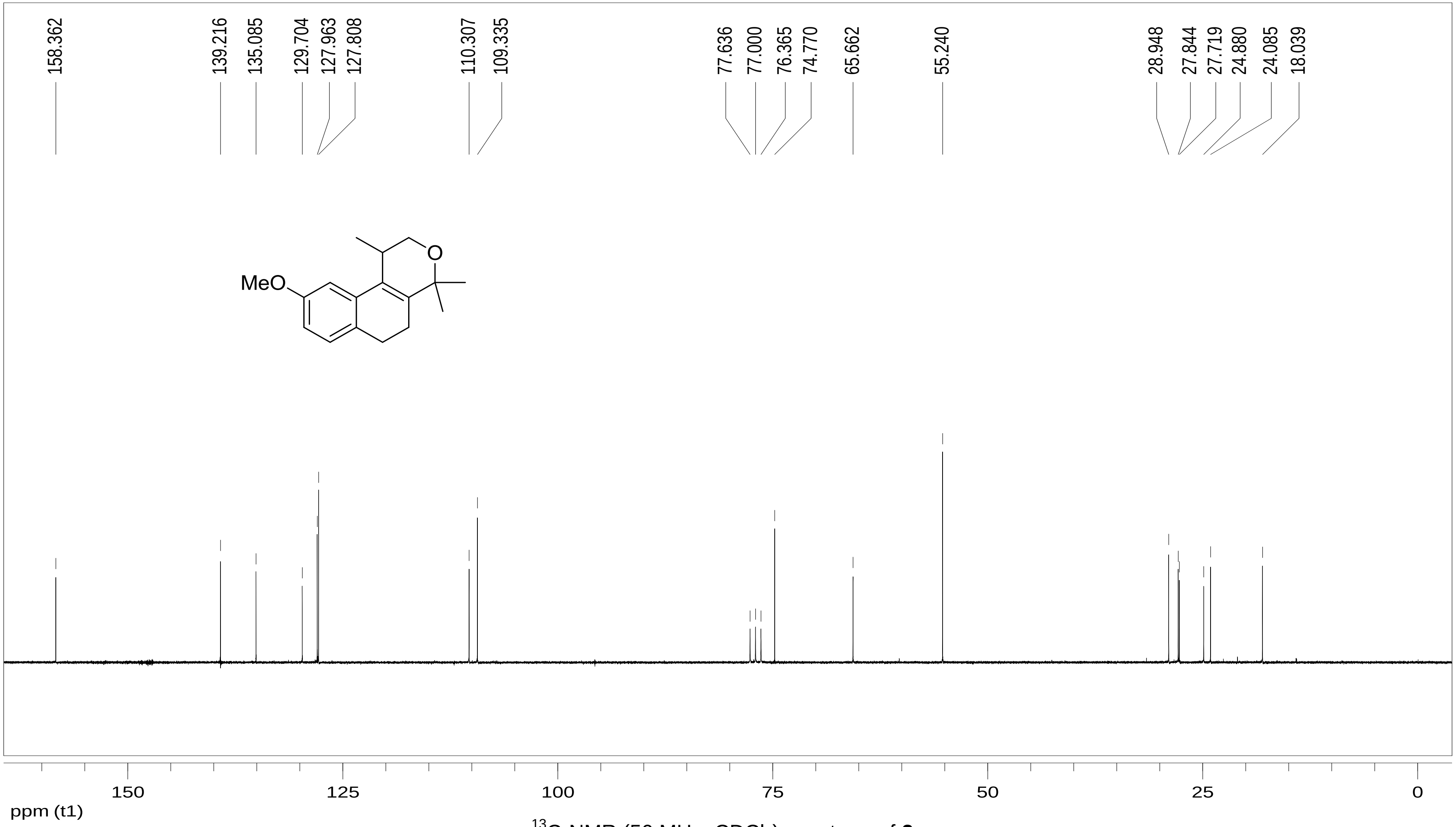

^{13}C-NMR (50 MHz, $CDCl_3$) spectrum of **2c**

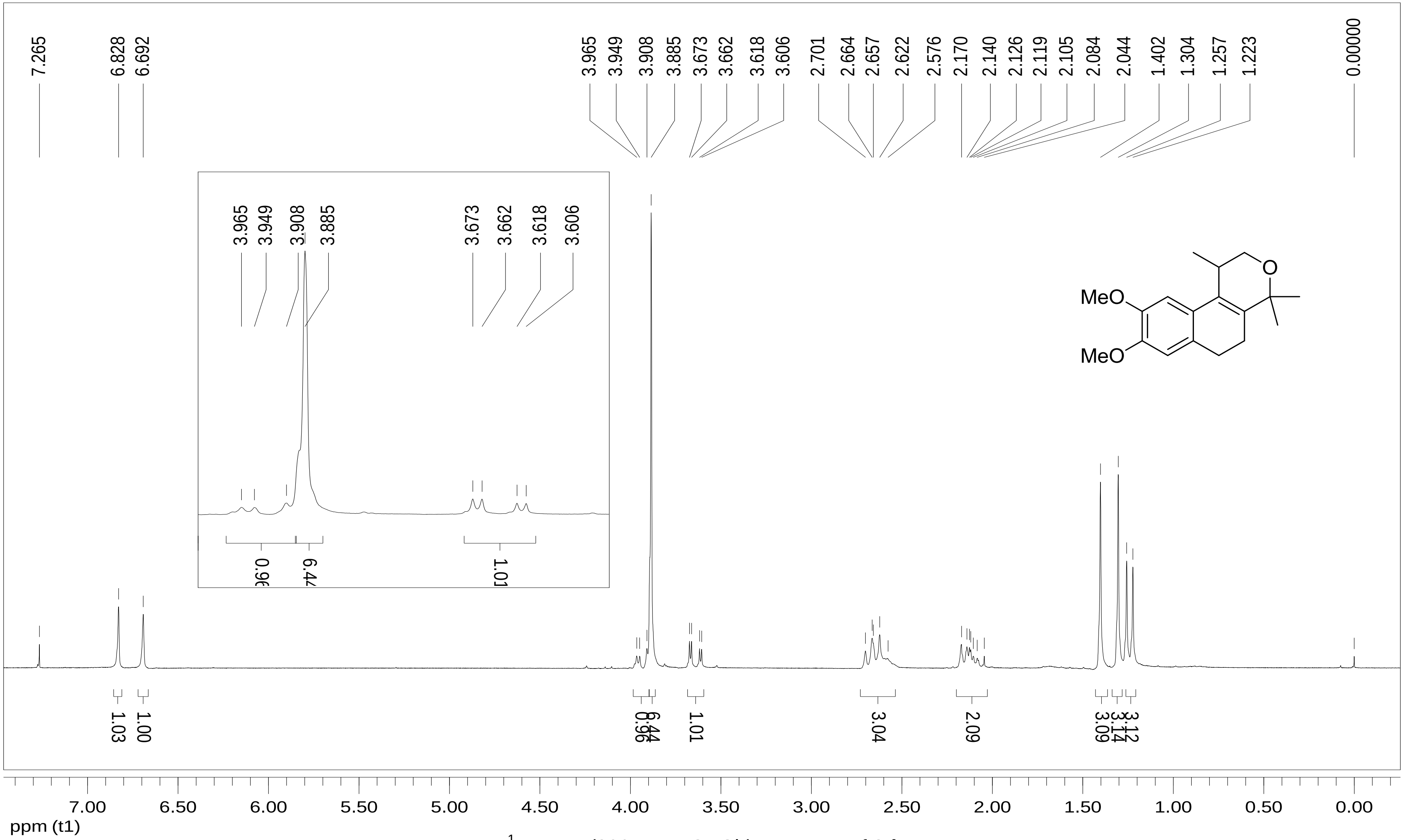

^{1}H-NMR (200 MHz, $CDCl_3$) spectrum of **2d**

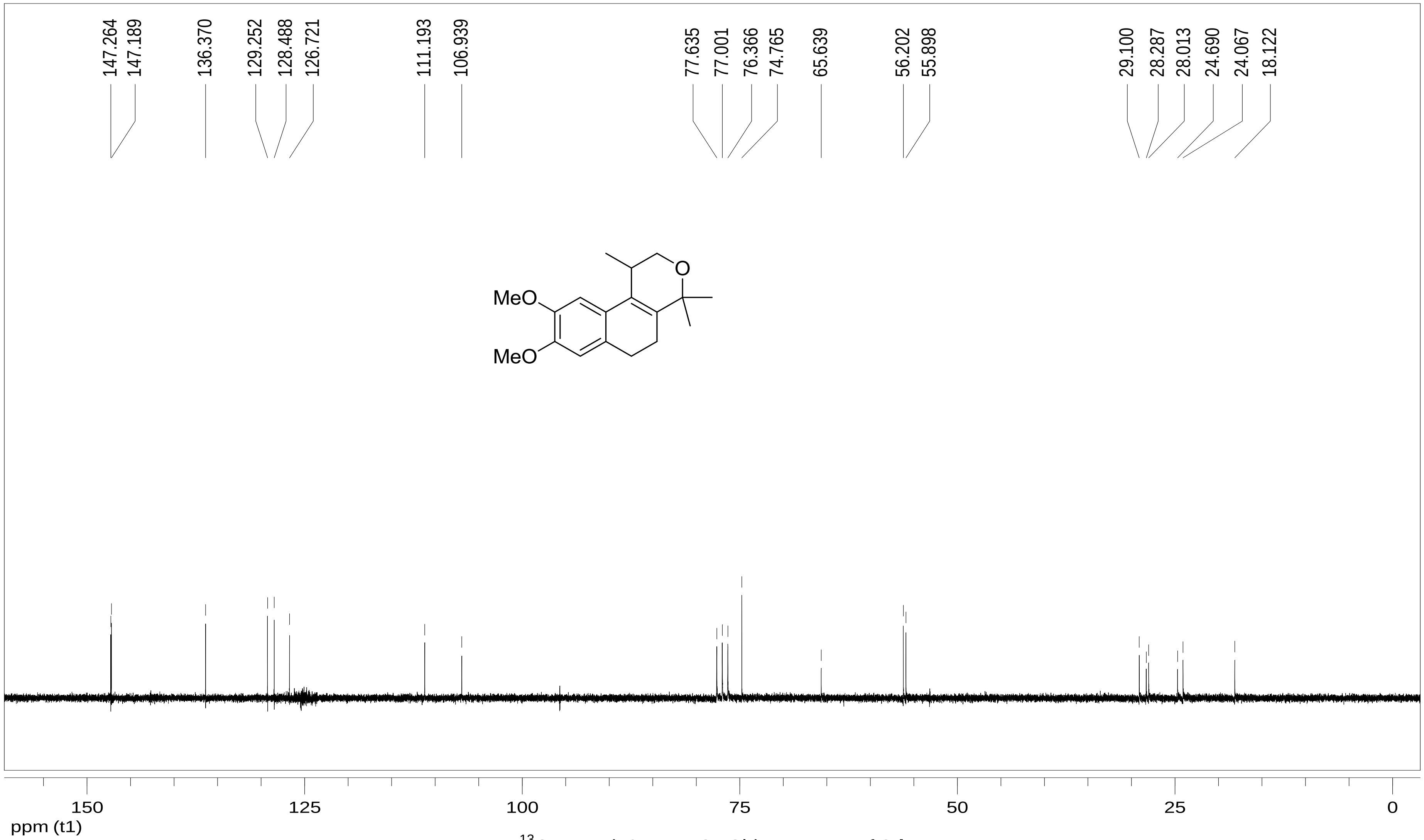

^{13}C-NMR (50 MHz, $CDCl_3$) spectrum of **2d**

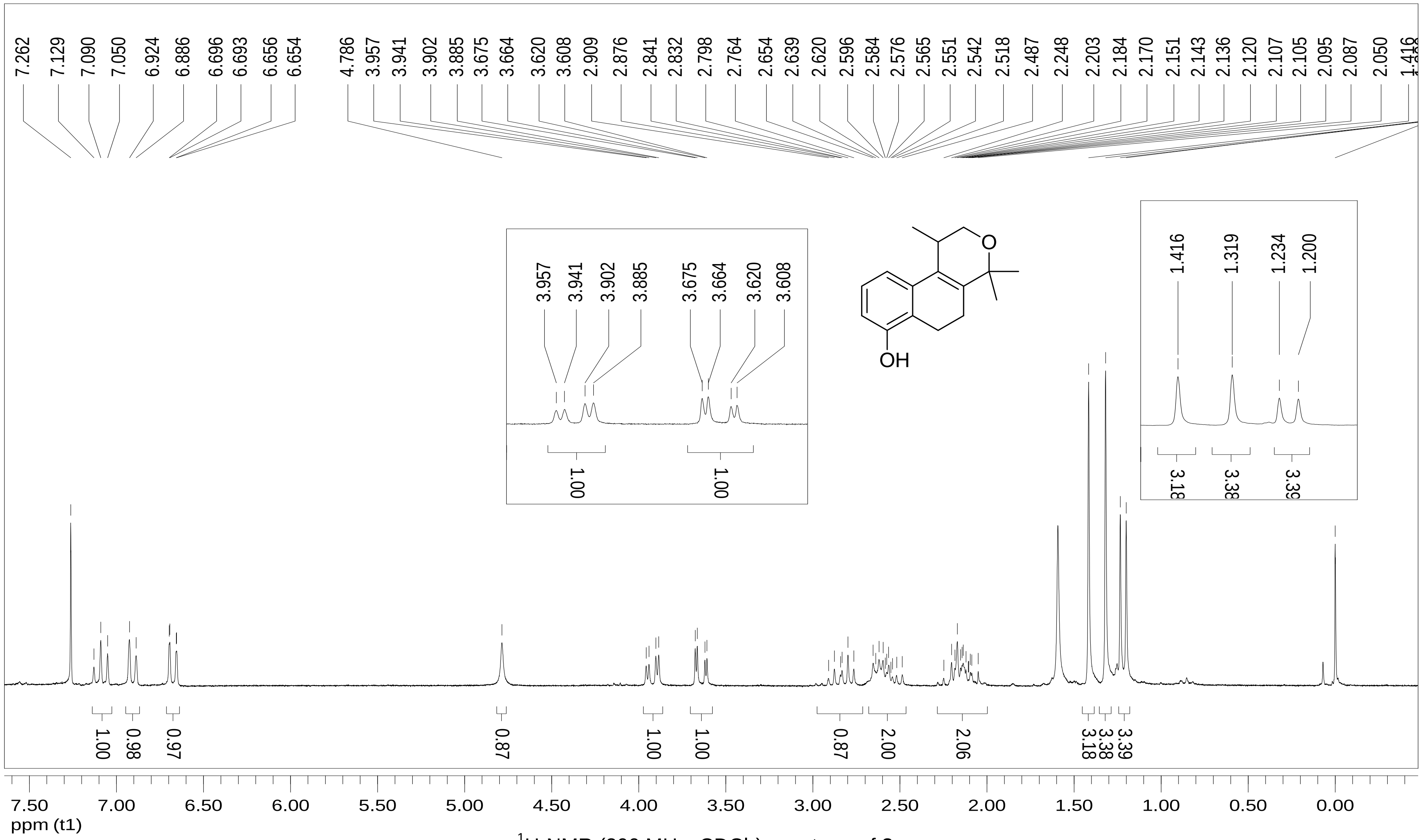

^{1}H-NMR (200 MHz, $CDCl_3$) spectrum of **3a**

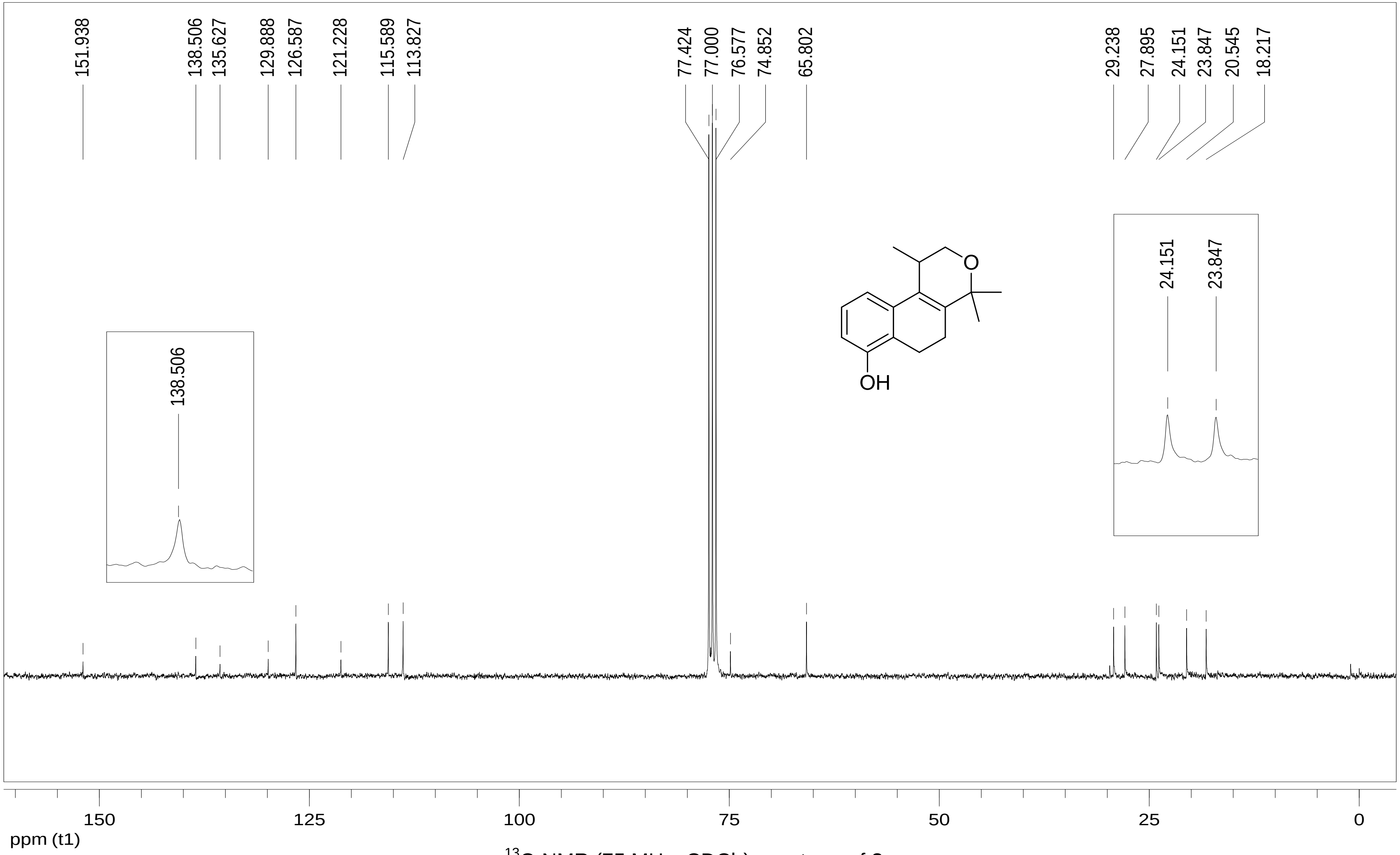

^{13}C-NMR (75 MHz, $CDCl_3$) spectrum of **3a**

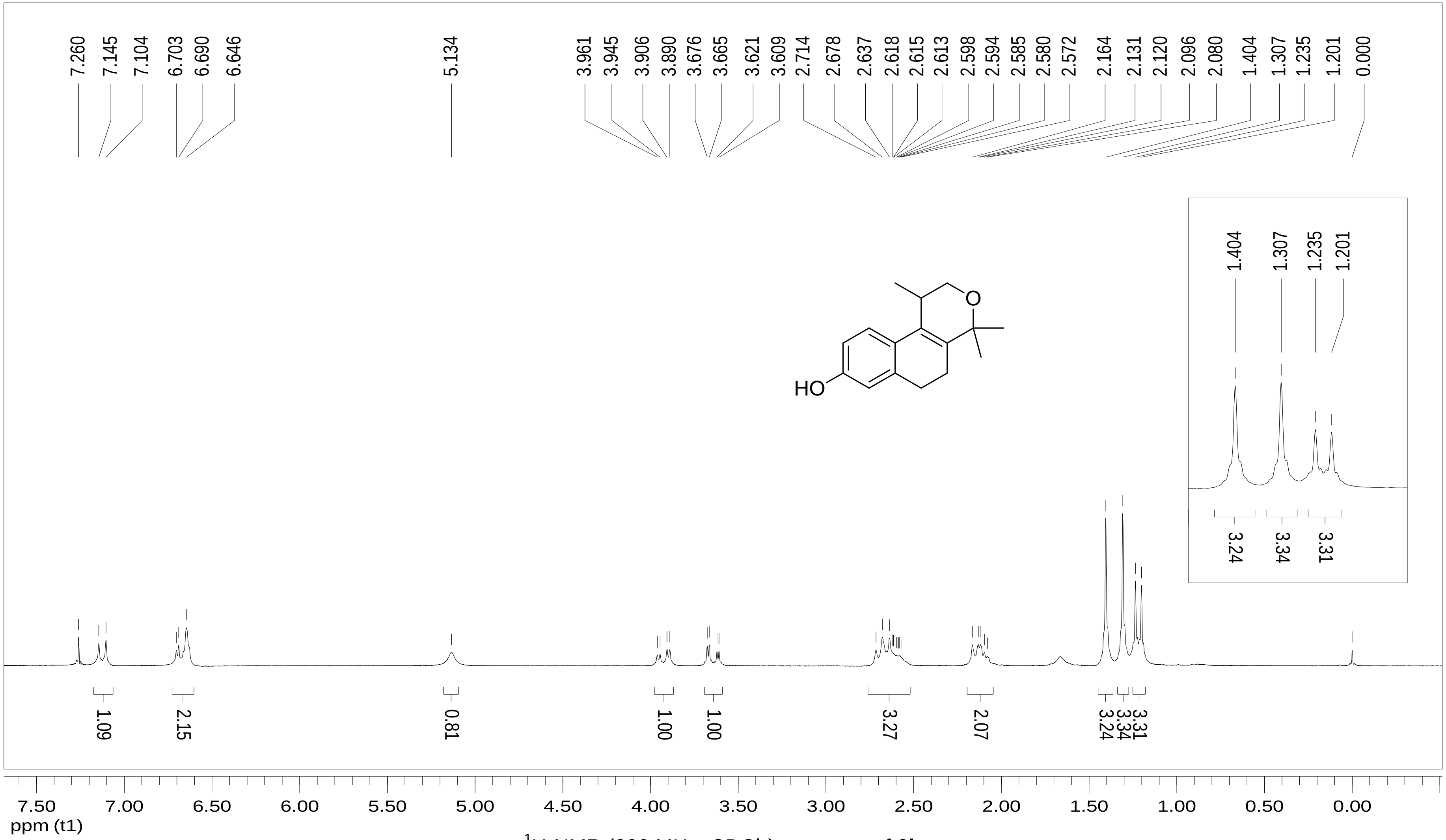

^{1}H-NMR (200 MHz, $CDCl_3$) spectrum of **3b**

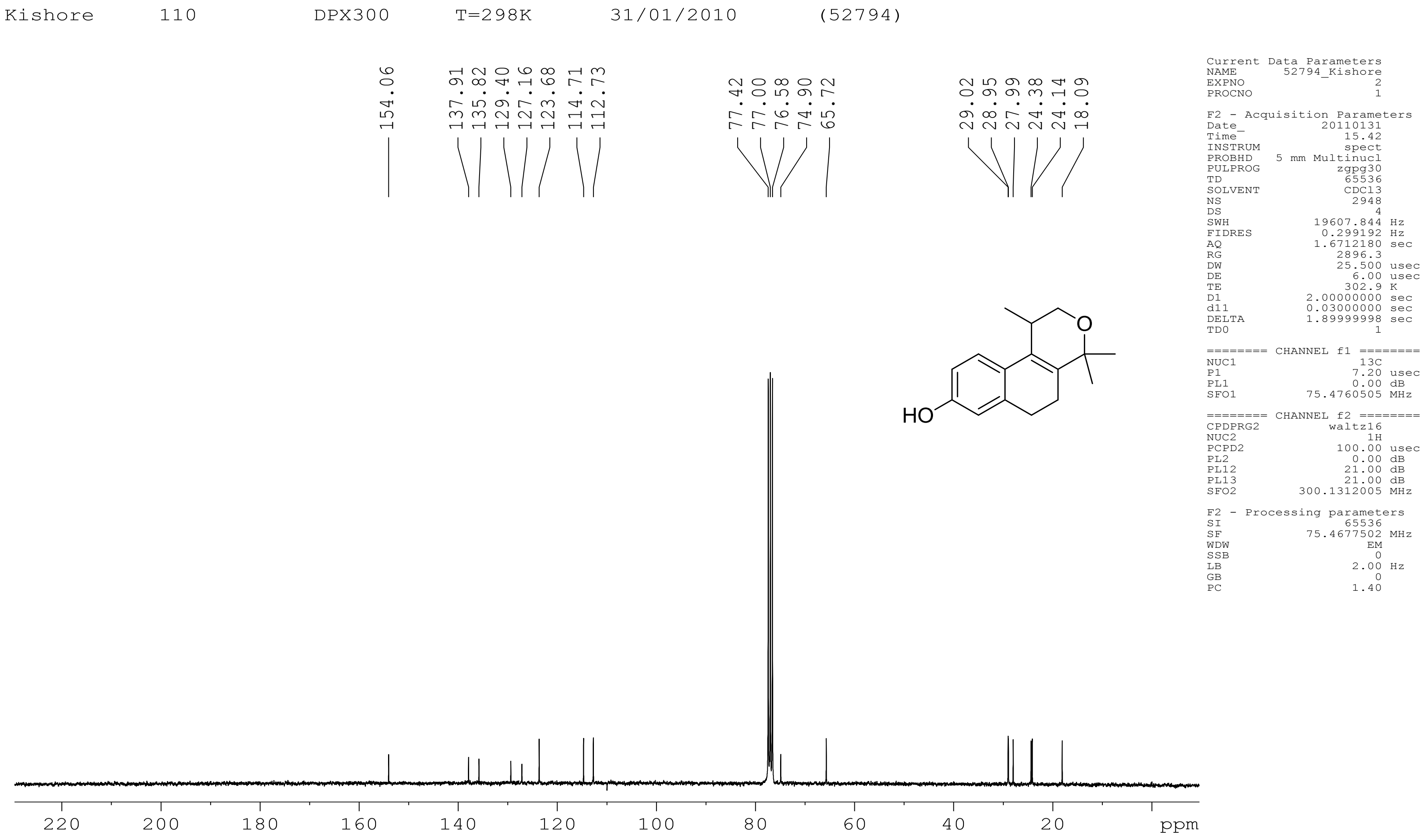

^{13}C-NMR (75 MHz, $CDCl_3$) spectrum of **3b**

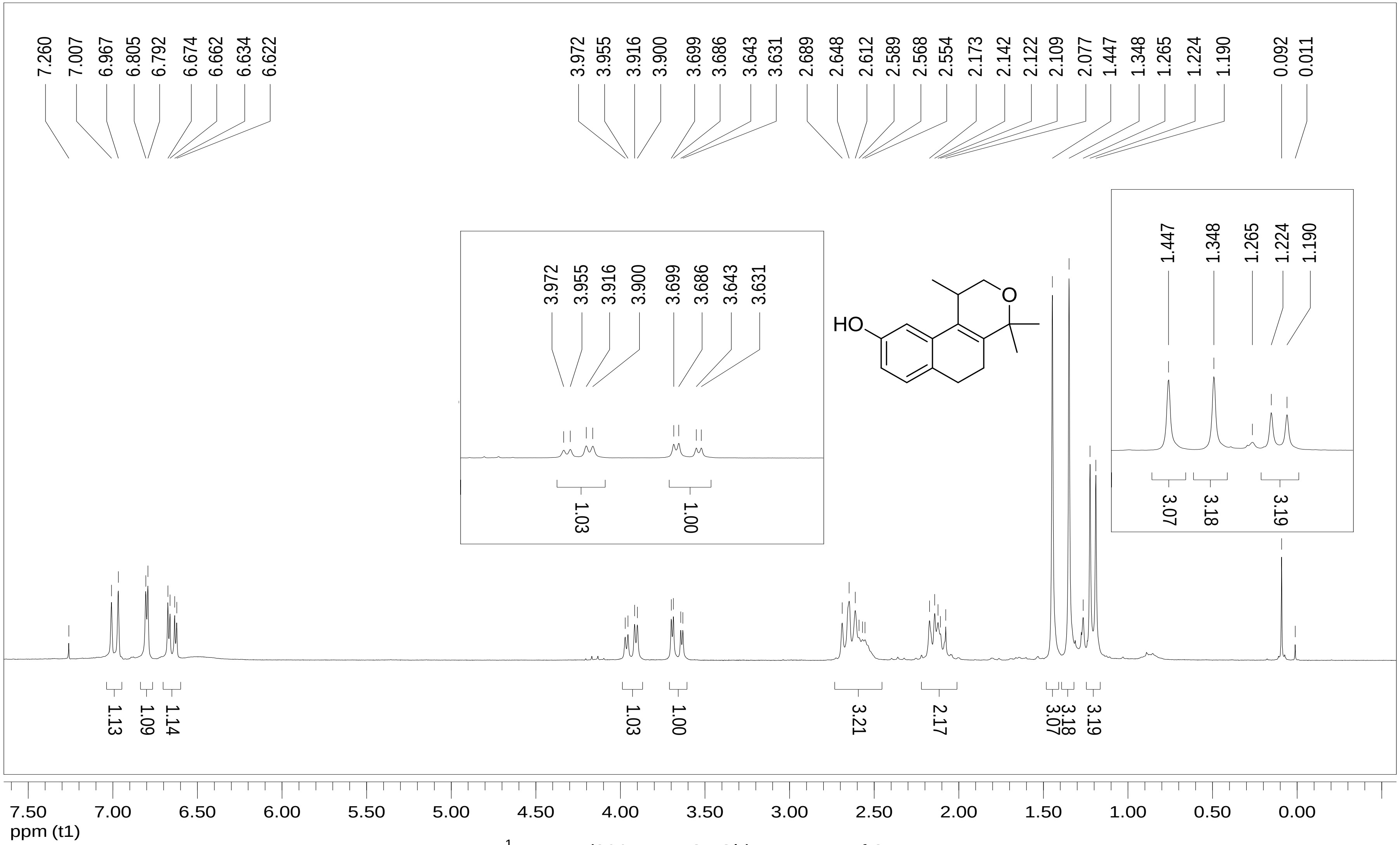

^{1}H-NMR (200 MHz, $CDCl_3$) spectrum of **3c**

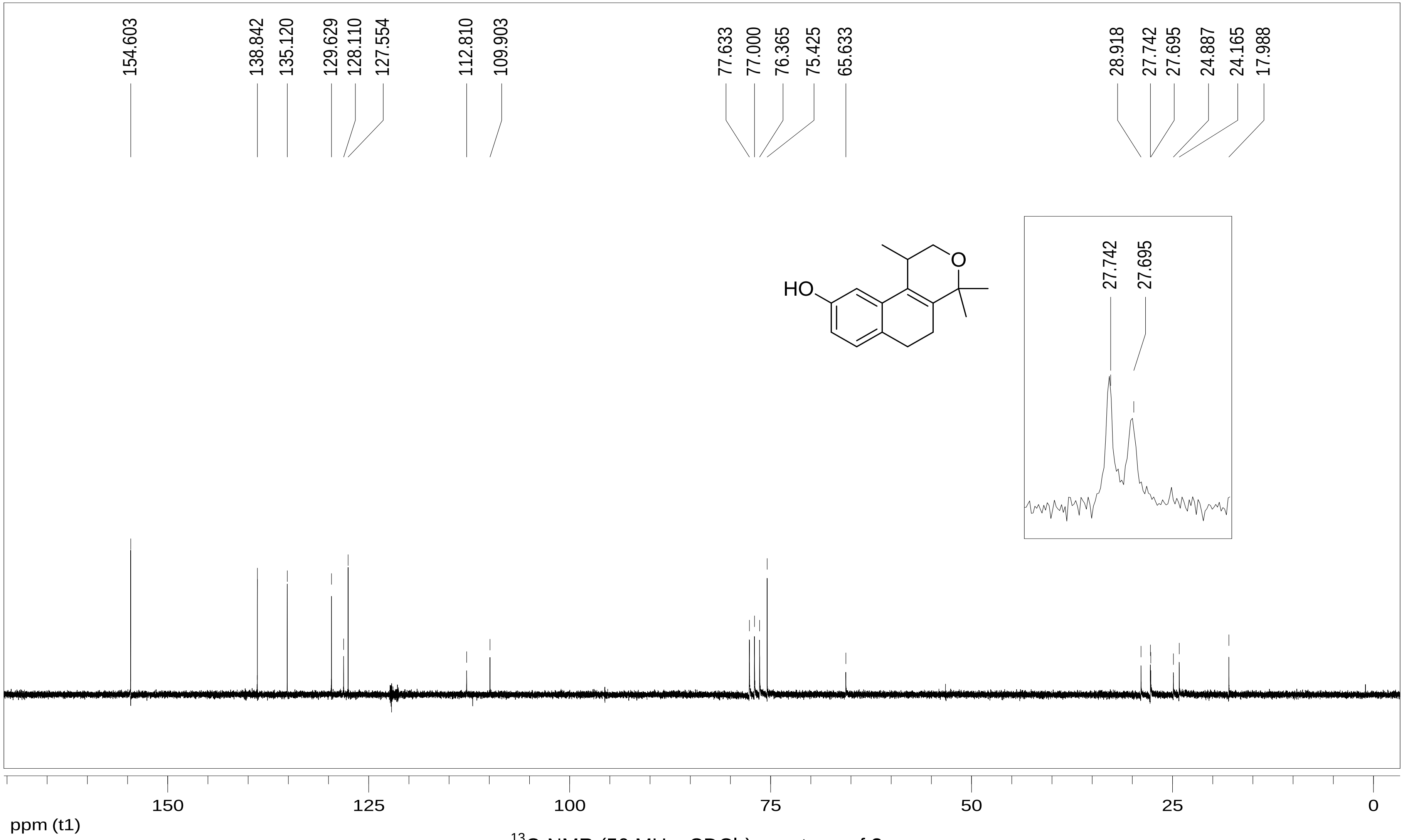

^{13}C-NMR (50 MHz, $CDCl_3$) spectrum of **3c**

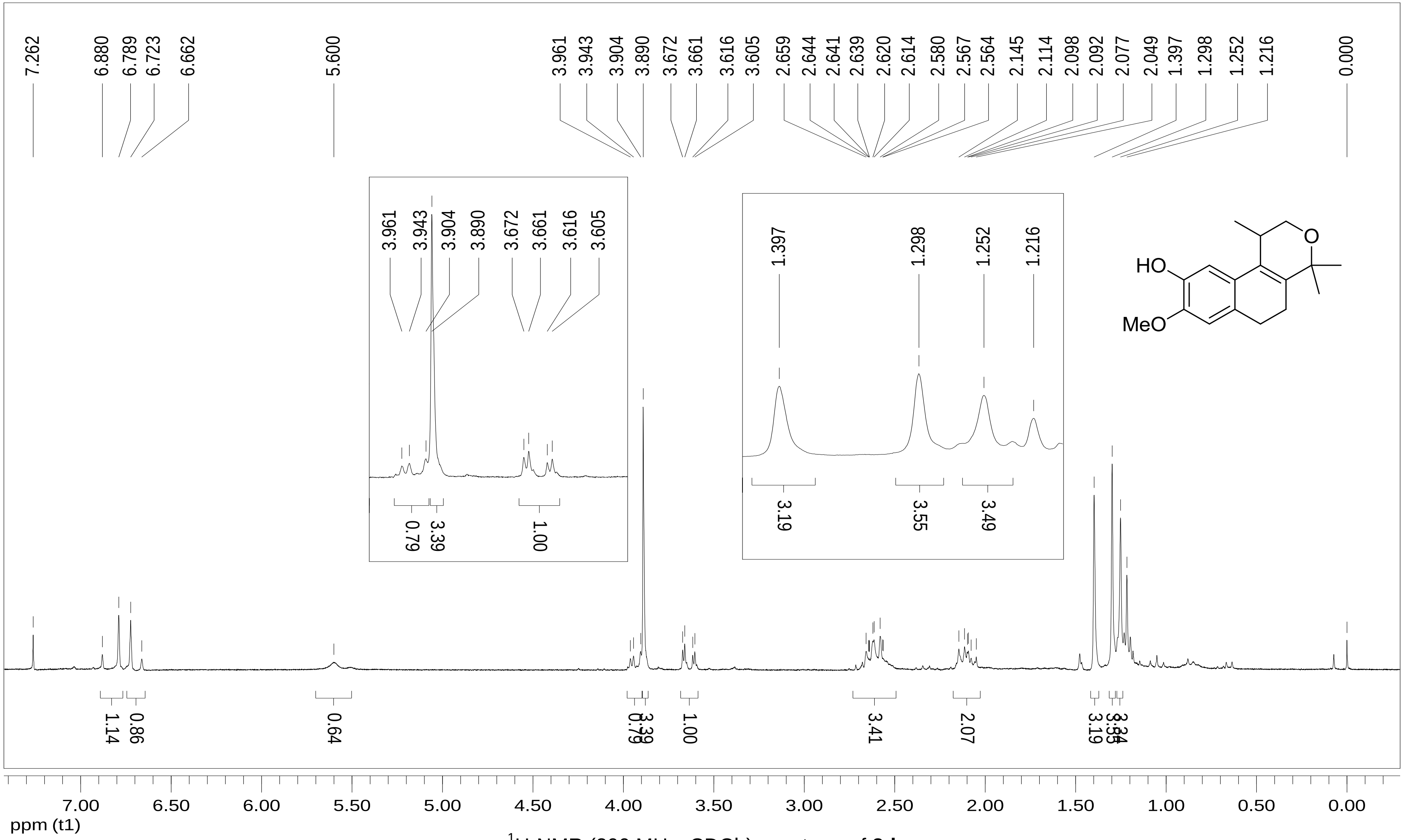

^{1}H-NMR (200 MHz, $CDCl_3$) spectrum of **3d**

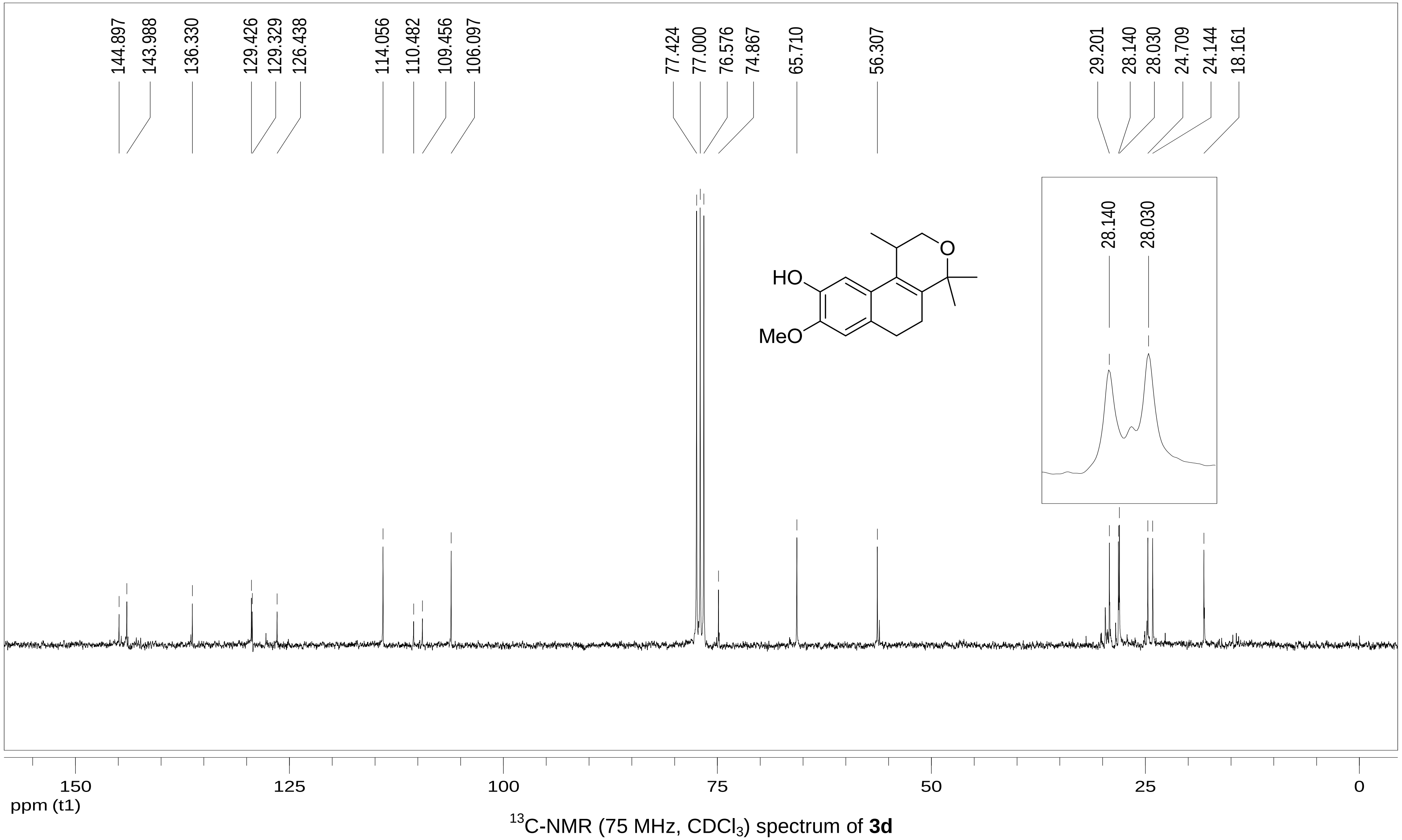

^{13}C-NMR (75 MHz, $CDCl_3$) spectrum of **3d**

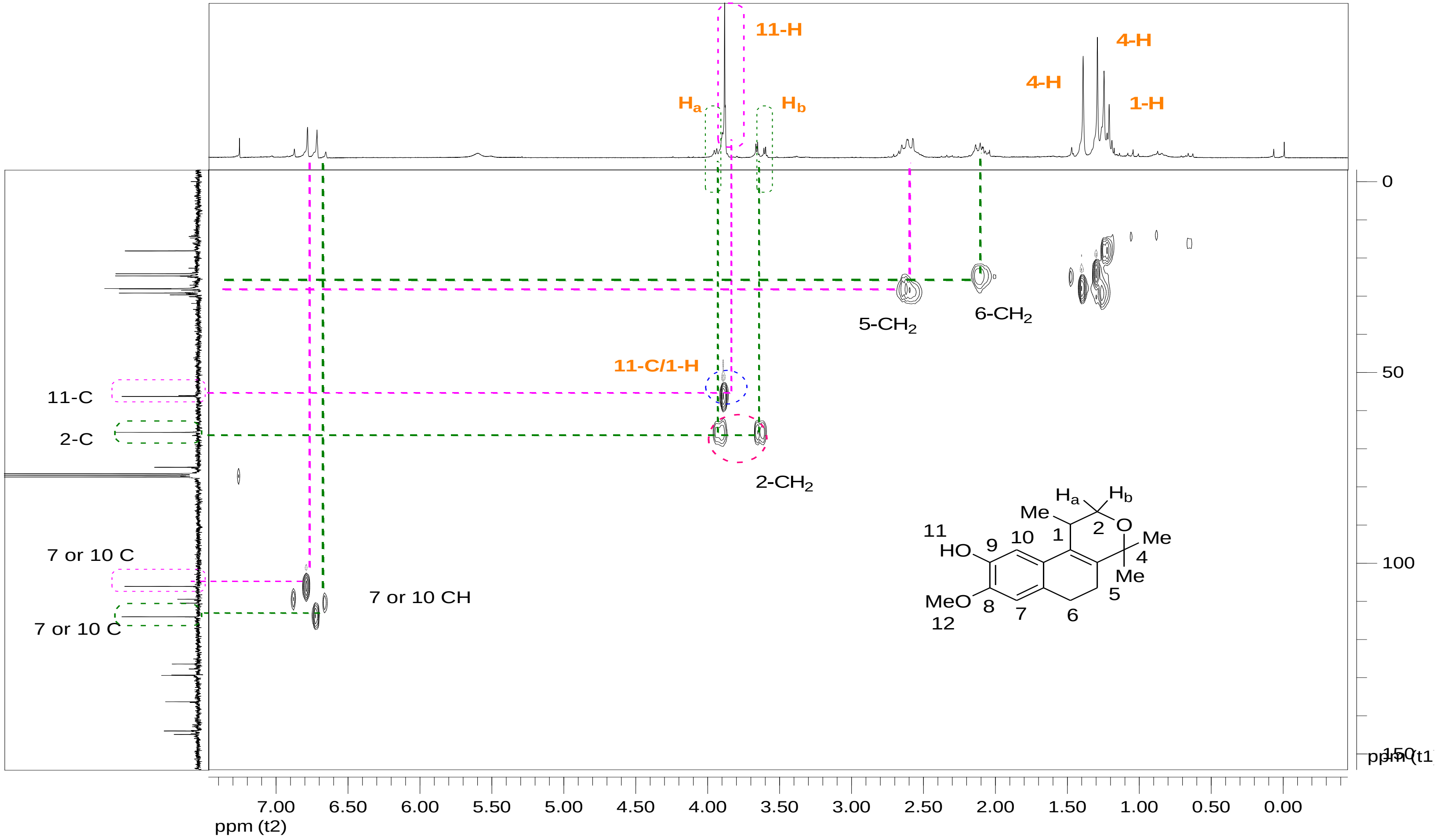

Selected HSQC (300 MHz, $CDCl_3$) correlated spectrum of **3d**

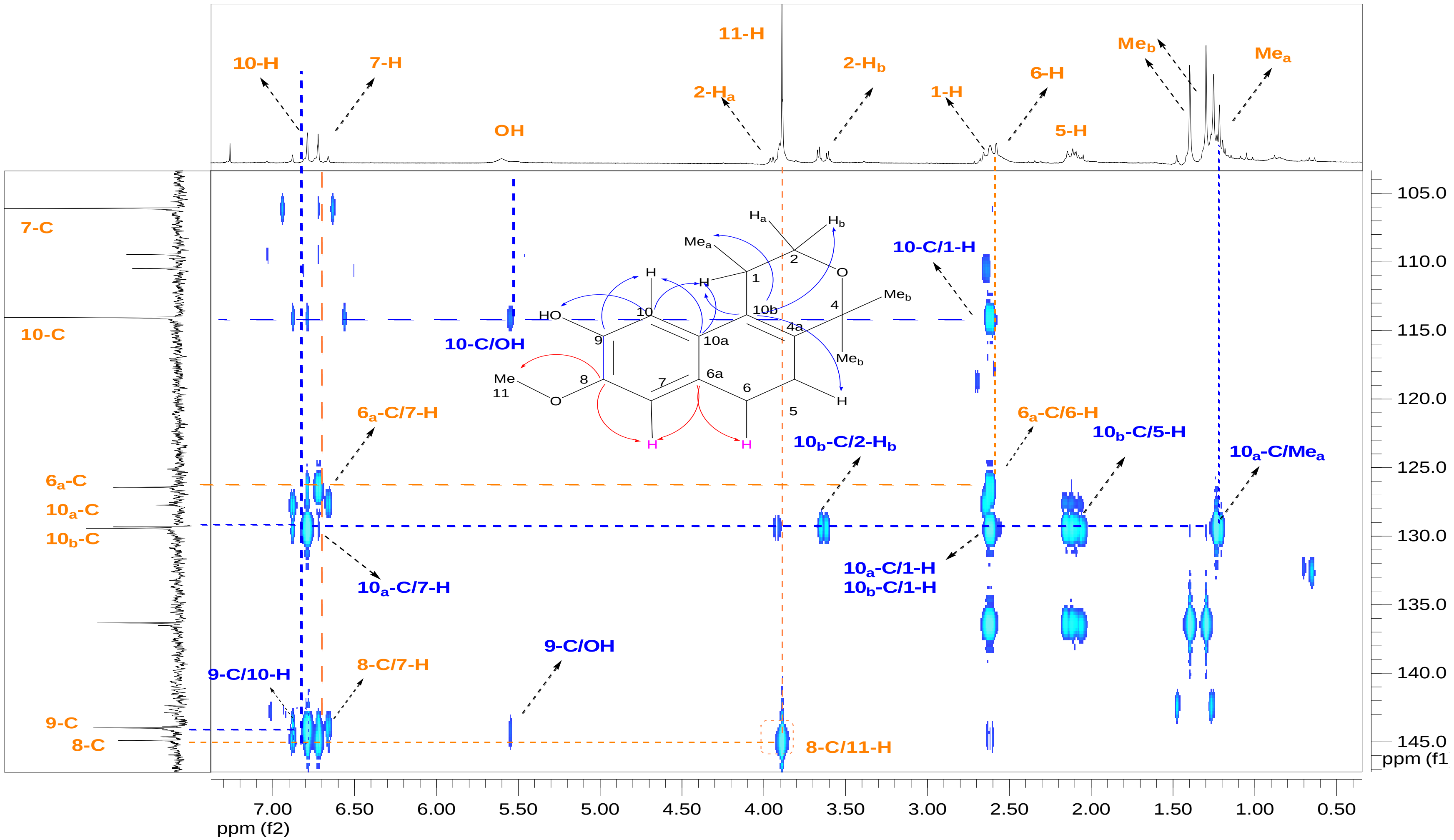

Selected HMBC (300 MHz, $CDCl_3$) correlated spectrum of **3d**

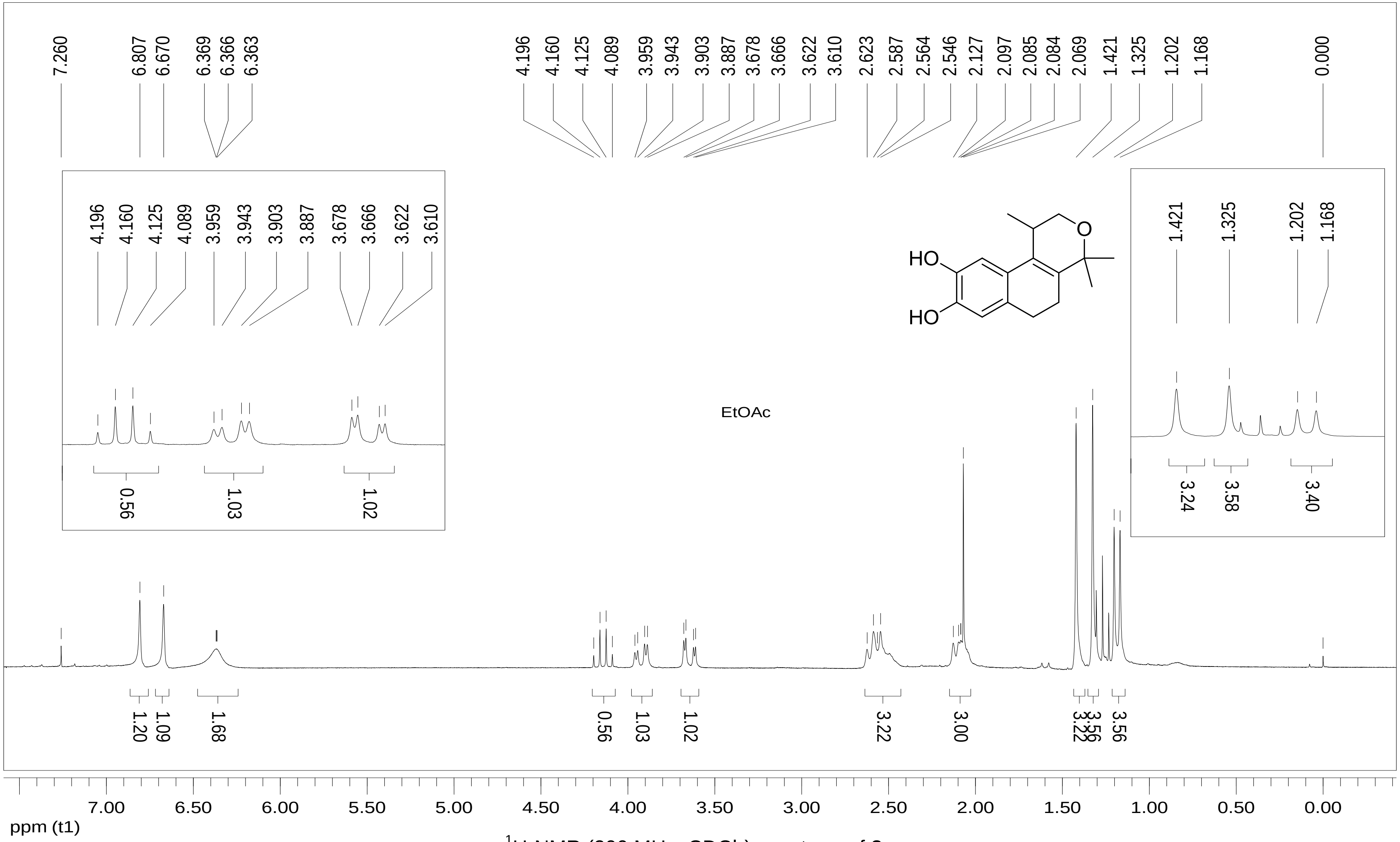

^{1}H-NMR (200 MHz, $CDCl_3$) spectrum of **3e**

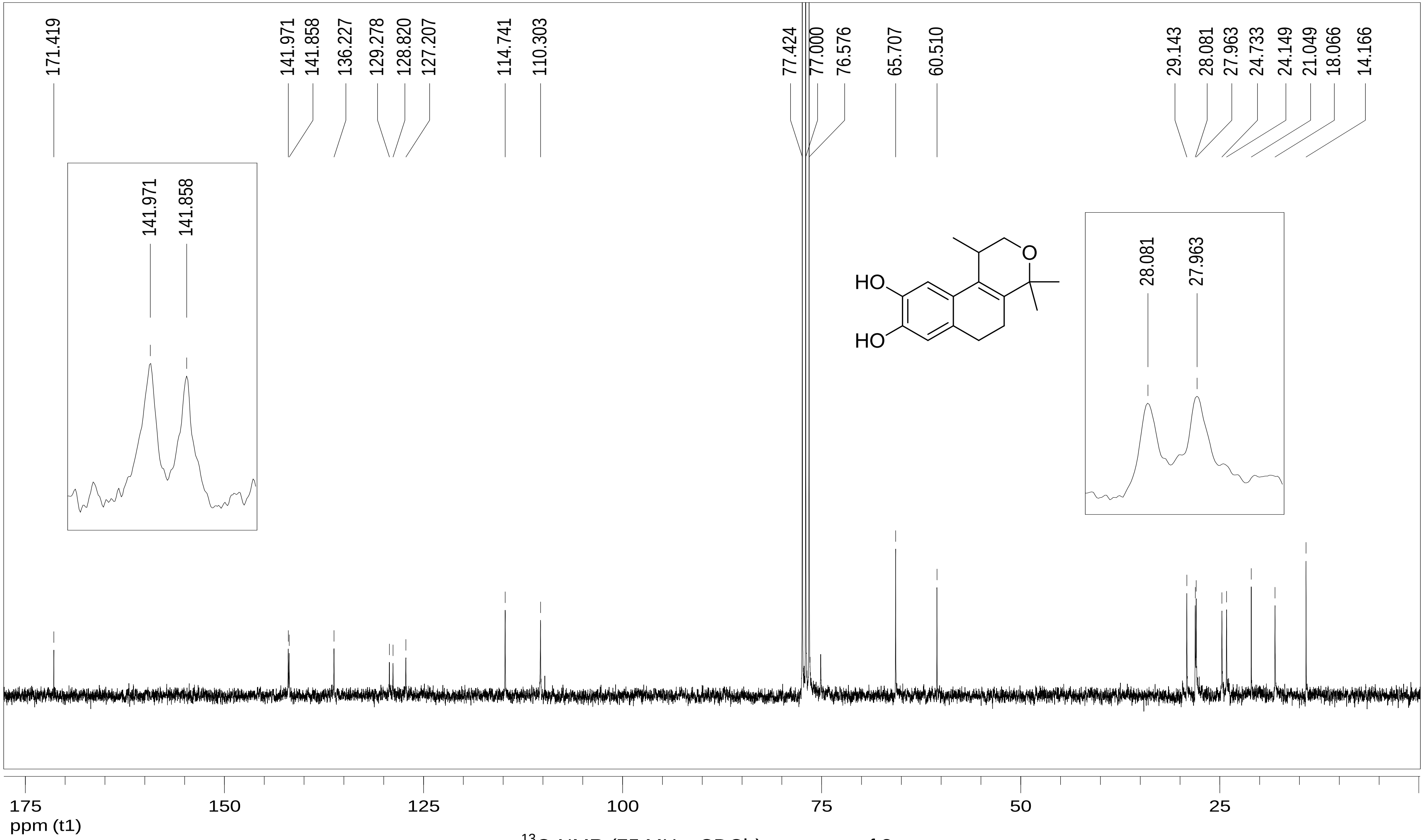

^{13}C-NMR (75 MHz, $CDCl_3$) spectrum of **3e**
